# Data on the cancer risk and mortalities induced by annual background radiations at various ages in Kohgiluyeh and Boyer-Ahmad province, Iran

**DOI:** 10.1016/j.dib.2020.105487

**Published:** 2020-04-18

**Authors:** Razzagh Abedi-Firouzjah, Hassan Vafapour, Amin Banaei, Kourosh Ebrahimnejad Gorji, Milad Najafzadeh, Gholamreza Ataei, Farideh Momeni

**Affiliations:** a Cellular and Molecular Research Center, Yasuj University of Medical Sciences, Yasuj, Iran; b Department of Medical Physics, Faculty of Medical Sciences, Tarbiat Modares University, Tehran, Iran; c Department of Radiology, Faculty of Paramedical Sciences, AJA University of Medical Sciences, Tehran, Iran; d Department of Medical Physics Radiobiology and Radiation Protection, Babol University of Medical Sciences, Babol, Iran; e Department of Radiology, Faculty of Para-Medicine, Hormozgan University of Medical Sciences, Bandare- Abbas, Iran; f Department of Radiology Technology, Faculty of Paramedical Sciences, Babol University of Medical Science, Babol, Iran; g Medical Physics and Medical Engineering Department, School of Medicine, Shiraz University of Medical Sciences, Shiraz, Iran

**Keywords:** Background radiation, Absorbed dose rate, Annual effective dose, Health risk estimation

## Abstract

Measurement of background radiations (BRs) as the sources of cancer risk, is important. The aim of this study was to measure the BR, as well as its cancer risk and mortalities in Kohgiluyeh and Boyer-Ahmad province (KBAp). Indoors and outdoors BRs were measured in eight cities utilizing a Geiger-Muller detector. Five main locations (north, east, west, south, and center) were chosen for measuring outdoor and indoor BRs in each city of KBAp. The BEIR VII-Phase 2 model was used to calculate the BRs induced cancer risks and mortalities of various cancer types at different ages. The average dose rates of outdoor and indoor were 136.9 ± 12.5 and 149.3 ± 19.8 nSv.h^−1^, respectively. The average annual effective doses (AEDs) for adults, children, and infants were 0.17, 0.19, and 0.22 mSv.y^−1^ due to the outdoor, and 0.73, 0.84, and 0.94 mSv.y^−1^ resulting from the indoor exposure, respectively. The average lifetime risk for one year BRs induced cancers was 164.8 ± 15.7 and 307.1 ± 32.3 (in 100,000 people) for new-borns male and female, in that order. This risk decreased with age and reached 11.2 ± 1.6 and 13.8 ± 1.6 (in 100,000 people) for men and women at the age of 80, respectively. The average lifetime risk of mortality due to cancers induced by annual BRs was 70.7 ± 8.3 and 113.8 ± 10.6 (incidence probability in 100,000 people) for new-borns male and female respectively. This risk decreased with age and reached 9.8 ± 1.3 and 12.2 ± 1.3 (in 100,000 people) for men and women at the age of 80 years, respectively.

Specifications table

**Table:** 

Subject	Radiation, Cancer research
Specific subject area	Estimating the risk of various cancers and mortalities induced by annual background radiations in eight cities of KBAp, Iran
Type of data	Tables
How data were acquired	BRs were measured in eight cities utilizing a Geiger-Muller detector. The BEIR VII-Phase 2 model was used to calculate the annual induced cancer risks and mortalities of various types of cancers in different ages. Instrument: A Geiger-Muller detector (GRAETZ X5C plus, Germany)
Data format	Raw, Analyzed.
Parameters for data collection	All measurements were made during daylight from October to December 2018, between 13:00 and 16:00. Five main areas (north, east, west, south, and center) were selected for measuring outdoor and indoor BRs in each city. Thirty buildings (6 buildings in each area) were randomly selected in each city to collect indoor measurements. Furthermore, five stations in each main area were randomly selected to measure outdoor BRs. The outdoor measurements were accomplished at least six meters away from any buildings and one meter above the ground surface.
Description of data collection	Eight cities were chosen for measurements. Indoor and outdoor BRs dose rates were measured for each city by the Geiger-Muller detector. The average (for each city) value was used to calculate the exposure rate of gamma BRs. For each measurement, the total exposure time was 30 min. The BEIR VII-Phase 2 model was used to calculate the induced annual cancer risks and various types of mortalities in different ages.
Data source location	Cities/Region: Eight cities in KBAp, including Yasuj, Dogonbadan, Dehdasht, Sisakht, Basht, Choram, Likak, and Landeh Country: Iran
Data accessibility	Raw and processed data are available with the article and in a supplementary file.

## Value of the data

• The data provide the lifetime cancer risk and mortalities of different cancer types induced by annual BRs in KBAp. Regarding the previous studies [1], [2], [3], [4], the BRs in this province are in the same ranges as many cities and regions in Iran. Therefore, these data are useful for estimating the cancer risks in this province and also in other regions of Iran.
• People living in/migrating to areas with the same BR, and also specialists in radiation sciences can benefit from these data.
• Future researches and experiments can use these data for studying the effects of BR on people's health. Furthermore, the radiation adaptivity in medium and high BR areas could be evaluated by combining our data with health situations (especially cancer risks) of local people in these regions.
A combination of our data and other similar studies gives a bright view of BRs in different areas of Iran and other countries. They will be very helpful for measuring BR health risks in people living at different regions.

## 1 Data description

Table 1 represents the BRs absorbed dose rates in KBAp. The corresponding AEDs (indoor and outdoor) for adults, children, and infants are shown in Table 2. For each of the selected areas, the mean and standard deviation (SD) of the measurements were calculated. Raw data of BRs measurement in each city could be found in supplementary materials.

**Table 1:** The cities altitude and the indoor and outdoor dose rate values due to BRs (nSv.h^−1^).

City	Altitude (m)	Range	Outdoor mean dose rate ± SD	Range	Indoor mean dose rate ± SD	indoors to outdoors ratio
Yasuj	1830	142–180	159.2 ± 14.1	145–198	169.6 ± 22.0	1.06 ± 0.09
Dogonbadan	725	115–165	135.4 ± 19.8	122–180	146.8 ± 21.1	1.09 ± 0.10
Dehdasht	806	115–147	124.2 ± 13.3	142–185	158.2 ± 17.1	1.27 ± 0.16
Sisakht	2230	110–185	150.4 ± 33.7	160–205	177.0 ± 20.2	1.22 ± 0.27
Basht	800	117–145	135.8 ± 10.9	142–150	143.8 ± 4.8	1.07 ± 0.12
Choram	740	115–148	131.0 ± 12.4	112–140	126.4 ± 10.7	0.96 ± 0.07
Likak	650	125–175	137.4 ± 21.4	115–185	153.8 ± 31.7	1.13 ± 0.23
Landeh	755	100–150	122.0 ± 20.2	100–160	121.4 ± 22.9	0.99 ± 0.08
**Average**	1067 ± 566	–	136.9 ± 12.5	–	149.3 ± 19.8	1.09 ± 0.14

**Table 2:** Mean ± SD values of AED for adults, children, and infants resulting from the indoor and outdoor BRs for eight cities (mSv.y^−1^).

City	Outdoors			Indoors		
	Adults	Children	Infants	Adults	Children	Infants
Yasuj	0.20 ± 0.02	0.22 ± 0.02	0.25 ± 0.02	0.83 ± 0.10	0.95 ± 0.12	1.07 ± 0.13
Dogonbadan	0.17 ± 0.02	0.19 ± 0.03	0.21 ± 0.03	0.72 ± 0.10	0.82 ± 0.12	0.93 ± 0.13
Dehdasht	0.15 ± 0.02	0.17 ± 0.02	0.20 ± 0.02	0.78 ± 0.08	0.89 ± 0.11	1.00 ± 0.11
Sisakht	0.18 ± 0.04	0.21 ± 0.05	0.24 ± 0.05	0.84 ± 0.11	0.96 ± 0.12	1.08 ± 0.13
Basht	0.17 ± 0.01	0.19 ± 0.02	0.21 ± 0.02	0.71 ± 0.02	0.81 ± 0.03	0.91 ± 0.03
Choram	0.16 ± 0.02	0.18 ± 0.02	0.21 ± 0.02	0.62 ± 0.05	0.71 ± 0.06	0.80 ± 0.07
Likak	0.17 ± 0.03	0.19 ± 0.03	0.22 ± 0.03	0.75 ± 0.16	0.86 ± 0.18	0.97 ± 0.20
Landeh	0.15 ± 0.02	0.17 ± 0.03	0.19 ± 0.03	0.60 ± 0.11	0.68 ± 0.13	0.77 ± 0.14
**Average**	0.17 ± 0.02	0.19 ± 0.02	0.22 ± 0.02	0.73 ± 0.09	0.83 ± 0.10	0.94 ± 0.11

Therefore, the values of lifetime risk of various cancers incidence and cancers mortalities (in 100,000 individuals) induced by annual BRs in KBAp averaged over all the mentioned cities were reported in Tables 3 and 4, respectively. These risks for each city were provided in tables as supplementary materials.

**Table 3:** Mean ± SD values of lifetime risk of various cancers (in 100,000 people) induced by annual BRs in KBAp averaged over all the mentioned cities.

	Age at exposure time (year)										
	0	5	10	15	20	30	40	50	60	70	80
Male											
Stomach	4.9 ± 0.6	4.2 ± 0.5	3.5 ± 0.5	3.0 ± 0.4	2.6 ± 0.3	1.8 ± 0.3	1.7 ± 0.2	1.6 ± 0.2	1.3 ± 0.2	0.9 ± 0.1	0.5 ± 0.1
Colon	21.6 ± 2.3	18.3 ± 1.9	15.5 ± 1.7	13.1 ± 1.3	11.1 ± 1.3	8.0 ± 1.0	7.8 ± 0.8	7.3 ± 0.7	6.0 ± 0.8	4.2 ± 0.5	1.9 ± 0.3
Liver	3.9 ± 0.5	3.2 ± 0.4	2.8 ± 0.3	2.3 ± 0.3	1.9 ± 0.3	1.4 ± 0.2	1.4 ± 0.2	1.2 ± 0.1	0.9 ± 0.1	0.5 ± 0.1	0.2 ± 0.0
Lung	20.2 ± 2.4	16.8 ± 1.9	13.9 ± 1.5	11.6 ± 1.0	9.6 ± 0.8	6.8 ± 0.7	6.7 ± 0.6	6.5 ± 0.5	5.7 ± 0.5	4.2 ± 0.3	2.2 ± 0.2
Prostate	6.0 ± 0.8	5.1 ± 0.6	4.3 ± 0.3	3.7 ± 0.3	3.1 ± 0.2	2.3 ± 0.2	2.3 ± 0.3	2.1 ± 0.2	1.7 ± 0.2	0.9 ± 0.1	0.3 ± 0.0
Bladder	13.4 ± 1.5	11.4 ± 1.3	9.6 ± 1.1	8.2 ± 0.9	6.9 ± 0.8	5.1 ± 0.6	5.1 ± 0.7	4.9 ± 0.5	4.2 ± 0.4	3.0 ± 0.4	1.5 ± 0.2
Other	72.2 ± 5.5	43.2 ± 4.1	32.3 ± 2.6	25.3 ± 2.2	20.1 ± 2.0	12.7 ± 1.5	11.1 ± 1.1	9.0 ± 1.0	6.3 ± 0.5	3.7 ± 0.5	1.5 ± 0.1
Thyroid	7.4 ± 0.8	4.9 ± 0.6	3.2 ± 0.4	2.1 ± 0.3	1.4 ± 0.1	0.6 ± 0.1	0.2 ± 0.0	0.1 ± 0.0	0.0 ± 0.0	0.0 ± 0.0	0.0 ± 0.0
All solids	149.5 ± 15.1	107.2 ± 11.3	85.2 ± 9.6	69.2 ± 7.3	56.6 ± 6.6	38.7 ± 4.5	36.3 ± 3.8	32.6 ± 3.5	26.2 ± 2.7	17.4 ± 1.6	8.1 ± 1.2
Leukemia	15.2 ± 1.7	9.6 ± 1.0	7.7 ± 0.8	6.8 ± 0.8	6.2 ± 0.7	5.4 ± 0.6	5.4 ± 0.6	5.4 ± 0.7	5.3 ± 0.6	4.7 ± 0.5	3.1 ± 0.4
All cancers	164.8 ± 15.7	116.7 ± 12.1	92.9 ± 10.8	76.0 ± 8.9	62.8 ± 7.0	44.1 ± 5.2	41.7 ± 5.4	38.0 ± 4.2	31.4 ± 3.7	22.1 ± 3.0	11.2 ± 1.6
Female											
Stomach	6.5 ± 0.7	5.5 ± 0.6	4.6 ± 0.6	3.9 ± 0.5	3.3 ± 0.3	2.3 ± 0.3	2.3 ± 0.3	2.1 ± 0.3	1.7 ± 0.3	1.2 ± 0.2	0.7 ± 0.1
Colon	14.1 ± 1.6	12.0 ± 1.3	10.2 ± 1.3	8.6 ± 0.9	7.3 ± 0.8	5.3 ± 0.7	5.1 ± 0.6	4.7 ± 0.6	4.0 ± 0.5	2.9 ± 0.4	1.5 ± 0.1
Liver	1.8 ± 0.2	1.5 ± 0.2	1.3 ± 0.2	1.0 ± 0.2	0.9 ± 0.1	0.6 ± 0.1	0.6 ± 0.1	0.6 ± 0.1	0.5 ± 0.1	0.3 ± 0.0	0.1 ± 0.0
Lung	47.1 ± 5.3	39.1 ± 4.5	32.4 ± 3.6	26.8 ± 3.1	22.2 ± 2.4	15.6 ± 2.0	15.4 ± 1.8	14.8 ± 1.6	12.9 ± 1.4	9.5 ± 1.0	5.0 ± 0.7
Breast	75.3 ± 6.8	58.8 ± 6.1	45.8 ± 5.3	35.6 ± 4.1	27.6 ± 3.8	16.3 ± 2.6	9.1 ± 1.7	4.5 ± 0.8	2.0 ± 0.3	0.8 ± 0.1	0.3 ± 0.0
Uterus	3.2 ± 0.4	2.7 ± 0.3	2.3 ± 0.2	1.9 ± 0.2	1.7 ± 0.2	1.2 ± 0.1	1.0 ± 0.1	0.8 ± 0.1	0.6 ± 0.1	0.3 ± 0.0	0.1 ± 0.0
Ovary	6.7 ± 0.6	5.5 ± 0.6	4.7 ± 0.5	3.9 ± 0.5	3.2 ± 0.3	2.2 ± 0.3	2.0 ± 0.2	1.6 ± 0.2	1.2 ± 0.1	0.7 ± 0.1	0.3 ± 0.0
Bladder	13.6 ± 1.5	11.6 ± 1.3	9.8 ± 1.0	8.3 ± 0.8	7.0 ± 0.8	5.1 ± 0.6	5.0 ± 0.6	4.8 ± 0.6	4.1 ± 0.5	3.0 ± 0.4	1.5 ± 0.2
Other	86.1 ± 9.1	46.2 ± 5.3	33.6 ± 3.8	26.3 ± 3.1	20.8 ± 2.7	13.3 ± 1.6	11.6 ± 1.4	9.5 ± 1.0	7.0 ± 0.9	4.4 ± 0.6	1.9 ± 0.3
Thyroid	40.8 ± 3.7	26.9 ± 2.3	17.7 ± 1.5	11.4 ± 1.0	7.3 ± 0.5	2.6 ± 0.3	0.9 ± 0.1	0.3 ± 0.0	0.1 ± 0.0	0.0 ± 0.0	0.0 ± 0.0
All solids	295.2 ± 25.1	209.9 ± 18.7	162.3 ± 15.1	127.8 ± 11.0	101.3 ± 10.6	64.4 ± 7.4	53.0 ± 5.1	43.6 ± 3.8	34.0 ± 3.4	23.0 ± 2.6	11.4 ± 1.3
Leukemia	11.9 ± 1.3	7.2 ± 0.8	5.5 ± 0.5	4.9 ± 0.5	4.6 ± 0.5	4.1 ± 0.5	4.0 ± 0.4	4.0 ± 0.4	3.7 ± 0.4	3.3 ± 0.4	2.4 ± 0.3
All cancers	307.1 ± 32.3	217.1 ± 24.6	167.9 ± 18.6	132.7 ± 15.1	105.8 ± 13.4	68.5 ± 7.8	57.0 ± 6.4	47.6 ± 5.5	37.7 ± 5.0	26.3 ± 3.3	13.8 ± 1.6

**Table 4:** Mean ± SD values of lifetime risk of various cancers mortalities (in 100,000 people) induced by annual BRs in KBAp averaged over all the mentioned cities.

	Age at exposure time (year)										
	0	5	10	15	20	30	40	50	60	70	80
Male											
Stomach	2.6 ± 0.3	2.2 ± 0.3	1.9 ± 0.3	1.6 ± 0.2	1.4 ± 0.1	1.0 ± 0.1	1.0 ± 0.1	0.8 ± 0.1	0.7 ± 0.1	0.5 ± 0.1	0.3 ± 0.0
Colon	10.5 ± 1.2	8.9 ± 1.0	7.5 ± 0.8	6.4 ± 0.7	5.4 ± 0.7	3.9 ± 0.5	3.9 ± 0.4	3.7 ± 0.5	3.2 ± 0.4	2.3 ± 0.2	1.4 ± 0.1
Liver	2.8 ± 0.4	2.4 ± 0.4	2.0 ± 0.3	1.7 ± 0.2	1.5 ± 0.2	1.0 ± 0.2	1.0 ± 0.1	0.9 ± 0.1	0.8 ± 0.1	0.5 ± 0.0	0.3 ± 0.0
Lung	20.4 ± 2.6	17.0 ± 2.1	14.1 ± 1.7	11.7 ± 1.4	9.7 ± 1.1	6.9 ± 0.8	6.9 ± 0.7	6.7 ± 0.8	6.0 ± 0.7	4.6 ± 0.5	2.7 ± 0.3
Prostate	1.1 ± 0.2	1.0 ± 0.1	0.8 ± 0.1	0.6 ± 0.1	0.6 ± 0.1	0.5 ± 0.1	0.4 ± 0.0	0.5 ± 0.0	0.5 ± 0.1	0.5 ± 0.1	0.3 ± 0.0
Bladder	2.9 ± 0.3	2.4 ± 0.3	2.1 ± 0.2	1.7 ± 0.2	1.5 ± 0.2	1.1 ± 0.2	1.1 ± 0.1	1.1 ± 0.1	1.1 ± 0.2	1.0 ± 0.1	0.6 ± 0.1
Other	25.7 ± 2.9	16.4 ± 2.1	12.9 ± 1.7	10.4 ± 1.3	8.6 ± 1.1	6.0 ± 0.9	5.7 ± 0.7	5.0 ± 0.7	3.7 ± 0.5	2.3 ± 0.4	1.1 ± 0.3
All solids	66.1 ± 6.8	50.2 ± 5.4	41.2 ± 4.6	34.3 ± 3.9	28.5 ± 3.3	20.4 ± 2.7	19.9 ± 2.2	18.6 ± 2.4	15.8 ± 1.7	11.6 ± 1.4	6.6 ± 0.8
Leukemia	4.6 ± 0.6	4.6 ± 0.7	4.6 ± 0.6	4.5 ± 0.6	4.3 ± 0.5	4.1 ± 0.4	4.3 ± 0.4	4.6 ± 0.6	4.7 ± 0.5	4.4 ± 0.5	3.3 ± 0.4
All cancers	70.7 ± 8.3	54.8 ± 5.9	45.8 ± 5.0	38.8 ± 4.6	32.9 ± 3.7	24.5 ± 2.9	24.2 ± 2.6	23.1 ± 2.5	20.5 ± 2.3	16.1 ± 1.9	9.8 ± 1.3
Female											
Stomach	3.7 ± 0.4	3.1 ± 0.4	2.6 ± 0.3	2.2 ± 0.3	1.9 ± 0.3	1.4 ± 0.2	1.3 ± 0.2	1.2 ± 0.1	1.0 ± 0.1	0.8 ± 0.1	0.5 ± 0.1
Colon	6.6 ± 0.8	5.5 ± 0.6	4.7 ± 0.6	4.0 ± 0.5	3.4 ± 0.3	2.4 ± 0.3	2.4 ± 0.4	2.3 ± 0.3	2.0 ± 0.3	1.6 ± 0.2	1.0 ± 0.2
Liver	1.5 ± 0.2	1.3 ± 0.1	1.1 ± 0.2	0.9 ± 0.1	0.8 ± 0.1	0.6 ± 0.1	0.5 ± 0.0	0.5 ± 0.1	0.5 ± 0.1	0.3 ± 0.0	0.2 ± 0.0
Lung	41.3 ± 4.5	34.3 ± 3.7	28.4 ± 3.2	23.6 ± 2.5	19.6 ± 2.2	13.7 ± 1.6	13.6 ± 1.7	13.1 ± 1.5	11.8 ± 1.4	9.0 ± 1.1	5.2 ± 0.7
Breast	17.6 ± 2.0	13.8 ± 1.6	10.7 ± 1.1	8.4 ± 0.9	6.5 ± 0.7	3.9 ± 0.5	2.3 ± 0.4	1.2 ± 0.2	0.6 ± 0.1	0.3 ± 0.0	0.1 ± 0.0
Uterus	0.7 ± 0.1	0.6 ± 0.1	0.5 ± 0.1	0.5 ± 0.0	0.4 ± 0.1	0.3 ± 0.0	0.3 ± 0.0	0.2 ± 0.0	0.2 ± 0.0	0.1 ± 0.0	0.1 ± 0.0
Ovary	3.5 ± 0.5	2.9 ± 0.4	2.5 ± 0.3	2.2 ± 0.3	1.8 ± 0.2	1.3 ± 0.1	1.3 ± 0.2	1.2 ± 0.1	1.0 ± 0.1	0.6 ± 0.1	0.3 ± 0.0
Bladder	3.8 ± 0.5	3.3 ± 0.4	2.8 ± 0.4	2.3 ± 0.3	2.0 ± 0.3	1.5 ± 0.2	1.5 ± 0.2	1.4 ± 0.2	1.4 ± 0.2	1.2 ± 0.2	0.8 ± 0.1
Other	31.6 ± 3.4	18.5 ± 2.1	14.1 ± 1.7	11.5 ± 1.1	9.5 ± 1.1	6.6 ± 0.8	6.2 ± 0.7	5.5 ± 0.6	4.4 ± 0.5	3.0 ± 0.4	1.5 ± 0.2
All solids	110.4 ± 10.6	83.3 ± 9.2	67.6 ± 7.0	55.4 ± 5.7	45.7 ± 4.8	31.6 ± 3.1	29.3 ± 2.3	26.7 ± 2.4	22.8 ± 2.1	17.0 ± 1.5	9.8 ± 1.0
Leukemia	3.4 ± 0.5	3.3 ± 0.4	3.4 ± 0.4	3.3 ± 0.5	3.3 ± 0.4	3.3 ± 0.3	3.3 ± 0.4	3.5 ± 0.5	3.5 ± 0.4	3.3 ± 0.5	2.4 ± 0.3
All cancers	113.8 ± 10.6	86.6 ± 9.2	71.0 ± 8.8	58.8 ± 6.6	49.0 ± 5.1	34.8 ± 3.1	32.6 ± 3.6	30.2 ± 2.9	26.3 ± 2.7	20.4 ± 1.8	12.2 ± 1.3

## 2 Experimental design, materials and methods

### 3.1 Study area and measurement setup

**Fig. 1:**
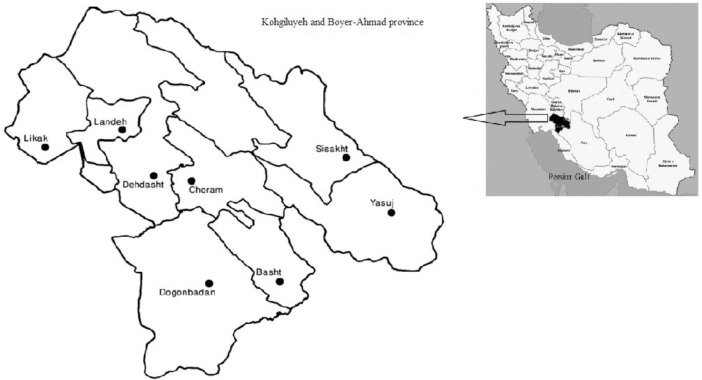
Map of Iran along with Kohgiluyeh and Boyer-Ahmad province.

The study area was KBAp. It is located in the southwest of Iran with an area equal to 16,249 km^2^. This province is a mountain and surrounded by Zagros Mountains with parallel strata. In addition, this province is situated at 49° 57′ and 50° 42′ east and 30° 9′ and 31° 32′ north (Fig. 1).

Eight cities (Yasuj, Dogonbadan, Dehdasht, Sisakht, Basht, Choram, Likak, Landeh) were chosen around the KBAp (Fig. 1) to determine the dose rate caused by the BR. A GPS system was used to find the position and altitude of each point in each of the cities. Indoor and outdoor BRs measurements were performed for each city by a calibrated Geiger-Muller detector (GRAETZ X5C plus, Strahlungsmeßtechnik GmbH, Germany). The detector was calibrated using the ^137^Cs in Iran Secondary Standard Dosimetry Laboratory (ISSDL).

**Fig. 2:**
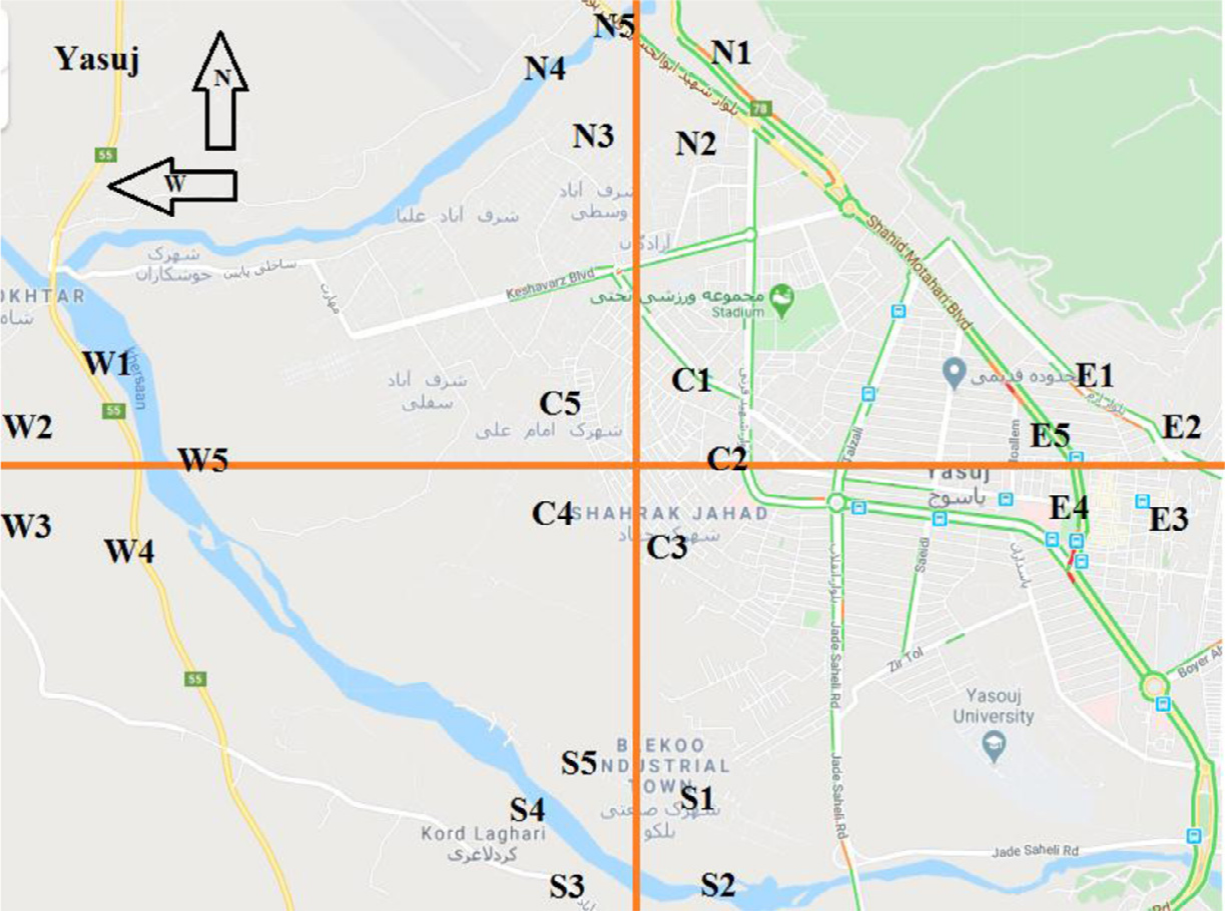
Five main locations (north, east, west, south, and center) along with five randomly stations of Yasuj city (as an example) for measuring outdoor background radiation.

All measurements were made during daylight from October to December 2018. The exposure rate meter has a maximum response to environmental radiation between the hours of 13:00 and 16:00, for this reason, all measurements were obtained at these times [5]. Five main areas (north, east, west, south, and center) were selected for measuring outdoor and indoor BRs in each city. To each of these areas, five stations were randomly selected to measure outdoor BRs (Fig. 2). In addition, thirty buildings (6 buildings in each main area) were randomly chosen in each city to collect indoor measurements. The building materials for all of the cities were approximately similar and are stone or bricks with the same soil materials.

The outdoor measurements were accomplished at least six meters away from any buildings and one meter above the ground surface, to diminish the effects of buildings on the radiation measurements. For each measurement, the total exposure time was 30 min. Finally, measurements were recorded for each region and the average value was used to calculate the exposure rate of gamma BRs.

### 2.2 The AED and excess lifetime cancer risk

Absorbed gamma BRs were used to calculate the AED delivered to the people living in the above-mentioned areas. The AED values were calculated as follows [6]:

(1) $$D_{Indoors}=D_{In}\times OF\times T\times CC$$

(2) $$D_{Outdoors}=D_{Out}\times OF\times T\times CC$$

Where the *D_Indoors_* and *D_Outdoors_* are the AED (nSv.y^−1^). Besides, *D_In_* and *D_Out_* are the mean absorbed indoor and outdoor dose rates in air, respectively (nSv.h^−1^). *OFs* are the fractions of time that were spent indoors or outdoors, which are 0.8 and 0.2, in that order. *T* is the time converter from hour to year (8760 h), and CCs are conversion coefficients (adults: 0.7, children: 0.8 and infants: 0.9), reported by the UNSCEAR 2000 to convert the absorbed dose in the air to the effective dose in humans [7].

The lifetime attributable risk of cancer incidence and lifetime attributable risk of cancer mortality for the various site of cancers at different exposure ages were calculated based on the preferred model reported with the committee to assess health risks from exposure to low levels of ionizing radiation (BEIR VII Phase 2) [8]. In this report, a low dose limit, doses less than 100 mGy and a gradual dose limit of 0.1 mGy/min are defined. Moderating factors are considered for cancer type, gender, age at exposure, and time elapsed after exposure [6]. A threshold-free linear model was used to estimate solid tumors and a quadratic linear model was used to estimate the risk of leukemia. The dose and dose rate effectiveness factor (DDREF) factor of 1.5 was applied for extrapolating risks from high dose/high dose rate exposures to low dose/low dose rate exposures. This means that the risk per Gy at low doses and low dose rates is expected to be 1.5 times lower than that at high doses and high dose rates. The report uses an exponential multiple-risk estimation model of the natural risk frequency in the community. For estimating the cancer risk based on age at radiation time (between progressive and incremental models) a combination of progressive and incremental models has been used such that in some cancers such as thyroid, the progressive model was applied. In some other cancers such as breast cancer in women, the incremental model and the weighted mean of both methods were used to estimate cancer risk. In the expression of risk, the committee has finally presented the life attributed risk (LAR) [6].

These values are presented in two tables as lifetime attributable risk of cancer incidence and lifetime attributable risk of cancer mortality for the various sites of cancers at different exposure ages. These tables present the additional risk of different cancers and the total risk of all cancers for ages ranging from 0 to 80 years in both sexes for a dose of 0.1 Gy per 100,000 individuals.

The calculated annual mean dose in each city was used to estimate the lifetime attributable risk of cancer incidence and lifetime attributable risk of cancer mortality in each city in people receiving BRs for one year. The cancer incidence and cancer mortality risks average over all cities were calculated and presented as LARs in KBAp.

## Appendix Supplementary materials

Image, application 1

Image, application 2
