# Supplementary material for: Data on the cancer risk and mortalities induced by annual background radiations at various ages in Kohgiluyeh and Boyer-Ahmad province, Iran
Source: Data Brief. 2020 Apr 18;30:105487. doi: 10.1016/j.dib.2020.105487 (PMC7178478; doi:10.1016/j.dib.2020.105487)
Supplement: Supplementary file 1 [file mmc1.docx]

**Supplementary Materials**

**Average and ranges of selected city altitude**

| **City** | **Altitude (m)** | **Range** |  |
| --- | --- | --- | --- |
| Yasuj | 1830 | 1825-1950 |  |
| Dogonbadan | 725 | 721-729 |  |
| Dehdasht | 806 | 801-811 |  |
| Sisakht | 2230 | 2212-2291 |  |
| Basht | 800 | 798-804 |  |
| Choram | 740 | 739-742 |  |
| Likak | 650 | 645-655 |  |
| Landeh | 755 | 750-760 |  |

|  |  |  |
| --- | --- | --- |

**Raw data of outdoor measurements of background radiations for five main locations (north, east, west, south, and center) along with five randomly stations at selected cities in Kohgiluye and Boyer Ahmad province**

| **City** | **Outdoor BRs (nSv.h^-1^)** | | | | | | |
| --- | --- | --- | --- | --- | --- | --- | --- |
|  | **North** | **South** | | **East** | **West** | | **Center** |
| Yasuj | 162 (75, 95, 170, 220, 250) | 142 (90, 115, 140, 170, 195) | | 152 (85, 120, 155, 180, 220) | 160 (100, 120, 170, 188, 220) | | 180 (90, 130, 200, 210, 270) |
| Dogonbadan | 142 (85, 85, 150, 190, 200) | 120 (100, 115, 115, 130, 140) | | 135 (100, 125, 130, 150, 170) | 115 (100, 105, 110, 130, 130) | | 165 (110, 130, 175, 190, 220) |
| Dehdasht | 115 (80, 90, 120, 135, 150) | 117 (95, 100, 115, 135, 140) | | 147 (75, 110, 150, 180, 220) | 125 (100, 110, 130, 135, 150) | | 117 (85, 100, 120, 130, 150) |
| Sisakht | 110 (100, 105, 110, 115, 120) | 122 (95, 110, 115, 140, 150) | | 185 (100, 150, 170, 235, 270) | 155 (110, 130, 145, 190, 200) | | 180 (100, 160, 170, 190, 280) |
| Basht | 140 (90, 100, 140, 180, 190) | 117 (85, 100, 120, 130, 150) | | 145 (90, 120, 145, 170, 200) | 140 (80, 120, 140, 160, 200) | | 137 (90, 130, 130, 160, 175) |
| Choram | 115 (70, 80, 110, 155, 160) | 137 (95, 110, 127, 175, 180) | | 130 (100, 115, 125, 150, 160) | 125 (100, 100, 125, 150, 150) | | 148 (75, 95, 150, 200, 220) |
| Likak | 127 (75, 95, 130, 155, 180) | 125 (70, 100, 120, 155, 180) | | 135 (80, 95, 140, 170, 190) | 125 (100, 105, 120, 150, 150) | | 175 (120, 148, 175, 200, 230) |
| Landeh | 105 (70, 90, 105, 120, 140) | 125 (90, 110, 115, 150, 160) | | 100 (90, 90, 95, 117, 110) | 130 (110, 130, 130, 130, 150) | | 150 (100, 118, 160, 170, 200) |
|  | | |  | | |  | |

**Raw data of indoor measurements of background radiations for five main locations (north, east, west, south, and center) along with six randomly buildings at selected cities in Kohgiluye and Boyer Ahmad province**

| **City** | **Indoor BRs (nSv.h^-1^)** | | | | |
| --- | --- | --- | --- | --- | --- |
|  | **North** | **South** | **East** | **West** | **Center** |
| Yasuj | 180 (80, 150, 170, 180, 220, 280) | 145 (100, 120, 130, 160, 170, 190) | 175 (90, 150, 175, 195, 200, 240) | 150 (100, 110, 140, 150, 180, 220) | 198 (95, 150, 195, 200, 250, 300) |
| Dogonbadan | 148 (90, 130, 145, 150, 170, 205) | 122 (95, 100, 120, 125, 141, 150) | 140 (100, 120, 130, 140, 170, 180) | 144 (98, 110, 145, 158, 174,180) | 180 (120, 150, 170, 180, 220, 240) |
| Dehdasht | 142 (85, 115, 140, 142, 170, 200) | 162 (95, 120, 155, 160, 210, 230) | 157 (75, 110, 157, 160, 200, 240) | 185 (150, 170, 185, 185, 200, 220) | 145 (100, 125, 130, 145, 180, 190) |
| Sisakht | 180 (100, 130, 150, 180, 240, 280) | 160 (120, 150, 170, 160, 160, 200) | 185 (100, 140. 170, 213, 230, 260) | 155 (120, 145, 150, 155, 170, 190) | 205 (110, 150, 215,206, 250, 300) |
| Basht | 142 (95, 120, 140, 143, 165, 190) | 150 (100, 110, 150, 170, 170, 200) | 145 (100, 110, 140, 160, 170, 190) | 137 (85, 110, 138, 150, 150, 190) | 145 (100, 110, 145, 155, 170, 190) |
| Choram | 112 (74, 85,110, 117, 136, 150) | 140 (100, 110, 140, 150, 160, 180) | 120 (90, 110, 110, 120, 140, 150) | 130 (100, 105, 135, 135, 155, 160) | 130 (70, 80, 130, 140, 170, 190) |
| Likak | 115 (80, 90, 115, 115, 140, 150) | 182 (160, 188, 180, 182, 190, 190) | 127 (80, 90,126, 130, 160, 175) | 160 (120, 137, 150, 180, 174, 200) | 185 (150, 160, 185, 190, 205, 220) |
| Landeh | 100 (50, 80, 100, 110, 115, 150) | 117 (85, 90, 116, 120, 140, 150) | 110 (90, 100, 110, 120, 120, 120) | 120 (110, 115, 120, 120, 125, 130) | 160 (120, 130, 160, 160, 190, 200) |

**The values of lifetime risk of various cancers (in 100000 people) induced by annual BRs in Yasuj city**

| **Age at exposure time (year)** | | | | | | | | | | | |
| --- | --- | --- | --- | --- | --- | --- | --- | --- | --- | --- | --- |
|  | **0** | **5** | **10** | **15** | **20** | **30** | **40** | **50** | **60** | **70** | **80** |
| **Male** |  |  |  |  |  |  |  |  |  |  |  |
| **Stomach** | 5.6 | 4.8 | 4.0 | 3.4 | 2.9 | 2.1 | 2.0 | 1.8 | 1.5 | 1.0 | 0.5 |
| **Colon** | 24.7 | 21.0 | 17.7 | 15.0 | 12.7 | 9.2 | 9.0 | 8.3 | 6.9 | 4.8 | 2.2 |
| **Liver** | 4.5 | 3.7 | 3.2 | 2.6 | 2.2 | 1.6 | 1.5 | 1.4 | 1.0 | 0.6 | 0.2 |
| **Lung** | 23.1 | 19.2 | 15.9 | 13.2 | 11.0 | 7.7 | 7.7 | 7.4 | 6.5 | 4.8 | 2.5 |
| **Prostate** | 6.8 | 5.9 | 4.9 | 4.2 | 3.5 | 2.6 | 2.6 | 2.4 | 1.9 | 1.0 | 0.4 |
| **Bladder** | 15.4 | 13.0 | 11.0 | 9.3 | 7.9 | 5.8 | 5.8 | 5.6 | 4.9 | 3.5 | 1.7 |
| **Other** | 82.6 | 49.4 | 37.0 | 29.0 | 23.0 | 14.6 | 12.7 | 10.3 | 7.2 | 4.2 | 1.7 |
| **Thyroid** | 8.5 | 5.6 | 3.7 | 2.4 | 1.5 | 0.7 | 0.2 | 0.1 | 0.0 | 0.0 | 0.0 |
| **All solids** | 171.1 | 122.6 | 97.5 | 79.2 | 64.8 | 44.3 | 41.5 | 37.3 | 29.9 | 19.9 | 9.3 |
| **Leukemia** | 17.4 | 11.0 | 8.8 | 7.7 | 7.1 | 6.2 | 6.2 | 6.2 | 6.0 | 5.4 | 3.5 |
| **All cancers** | 188.6 | 133.6 | 106.3 | 87.0 | 71.9 | 50.5 | 47.7 | 43.5 | 36.0 | 25.2 | 12.8 |
|  |  |  |  |  |  |  |  |  |  |  |  |
| **Female** |  |  |  |  |  |  |  |  |  |  |  |
| **Stomach** | 7.4 | 6.3 | 5.3 | 4.5 | 3.8 | 2.6 | 2.6 | 2.4 | 2.0 | 1.4 | 0.8 |
| **Colon** | 16.2 | 13.8 | 11.6 | 9.9 | 8.4 | 6.0 | 5.8 | 5.4 | 4.6 | 3.3 | 1.7 |
| **Liver** | 2.1 | 1.7 | 1.5 | 1.2 | 1.0 | 0.7 | 0.7 | 0.7 | 0.5 | 0.4 | 0.1 |
| **Lung** | 53.9 | 44.7 | 37.1 | 30.7 | 25.5 | 17.8 | 17.7 | 16.9 | 14.8 | 10.8 | 5.7 |
| **Breast** | 86.2 | 67.2 | 52.4 | 40.7 | 31.6 | 18.6 | 10.4 | 5.2 | 2.3 | 0.9 | 0.3 |
| **Uterus** | 3.7 | 3.1 | 2.6 | 2.2 | 1.9 | 1.3 | 1.2 | 1.0 | 0.7 | 0.4 | 0.1 |
| **Ovary** | 7.7 | 6.5 | 5.4 | 4.4 | 3.7 | 2.5 | 2.3 | 1.8 | 1.3 | 0.8 | 0.4 |
| **Bladder** | 15.6 | 13.2 | 11.2 | 9.5 | 8.0 | 5.8 | 5.7 | 5.4 | 4.7 | 3.5 | 1.8 |
| **Other** | 98.5 | 52.9 | 38.5 | 30.1 | 23.8 | 15.2 | 13.3 | 10.9 | 8.0 | 5.0 | 2.2 |
| **Thyroid** | 46.6 | 30.8 | 20.2 | 13.1 | 8.3 | 3.0 | 1.0 | 0.3 | 0.1 | 0.0 | 0.0 |
| **All solids** | 337.8 | 240.2 | 185.8 | 146.3 | 115.9 | 73.7 | 60.6 | 49.9 | 38.9 | 26.3 | 13.0 |
| **Leukemia** | 13.6 | 8.2 | 6.3 | 5.6 | 5.2 | 4.6 | 4.6 | 4.6 | 4.2 | 3.8 | 2.7 |
| **All cancers** | 351.5 | 248.5 | 192.1 | 151.9 | 121.1 | 78.4 | 65.2 | 54.4 | 43.1 | 30.1 | 15.7 |

**The values of lifetime risk of various cancers mortalities (in 100000 people) induced by annual BRs in Yasuj city**

| **Age at exposure time (year)** | | | | | | | | | | | | | | | | | | |
| --- | --- | --- | --- | --- | --- | --- | --- | --- | --- | --- | --- | --- | --- | --- | --- | --- | --- | --- |
|  | **0** | **5** | **10** | **15** | **20** | | **30** | | **40** | | **50** | | **60** | | **70** | | **80** | |
| **Male** |  |  |  |  |  | |  | |  | |  | |  | |  | |  | |
| **Stomach** | 3.0 | 2.5 | 2.2 | 1.8 | | 1.5 | | 1.2 | | 1.1 | | 1.0 | | 0.8 | | 0.6 | | 0.3 |
| **Colon** | 12.0 | 10.2 | 8.6 | 7.3 | | 6.2 | | 4.5 | | 4.4 | | 4.2 | | 3.6 | | 2.6 | | 1.5 |
| **Liver** | 3.2 | 2.7 | 2.3 | 2.0 | | 1.7 | | 1.2 | | 1.2 | | 1.0 | | 0.9 | | 0.6 | | 0.3 |
| **Lung** | 23.4 | 19.4 | 16.1 | 13.4 | | 11.1 | | 7.9 | | 7.9 | | 7.7 | | 6.8 | | 5.2 | | 3.1 |
| **Prostate** | 1.3 | 1.1 | 0.9 | 0.7 | | 0.7 | | 0.5 | | 0.4 | | 0.5 | | 0.5 | | 0.5 | | 0.4 |
| **Bladder** | 3.3 | 2.8 | 2.4 | 2.0 | | 1.7 | | 1.3 | | 1.3 | | 1.3 | | 1.3 | | 1.1 | | 0.7 |
| **Other** | 29.4 | 18.8 | 14.7 | 11.9 | | 9.9 | | 6.9 | | 6.5 | | 5.7 | | 4.3 | | 2.6 | | 1.3 |
| **All solids** | 75.6 | 57.5 | 47.2 | 39.2 | | 32.7 | | 23.3 | | 22.8 | | 21.3 | | 18.1 | | 13.3 | | 7.5 |
| **Leukemia** | 5.2 | 5.2 | 5.2 | 5.2 | | 4.9 | | 4.7 | | 4.9 | | 5.2 | | 5.4 | | 5.1 | | 3.8 |
| **All cancers** | 80.9 | 62.7 | 52.4 | 44.4 | | 37.6 | | 28.0 | | 27.7 | | 26.5 | | 23.5 | | 18.4 | | 11.3 |
|  |  |  |  |  | |  | |  | |  | |  | |  | |  | |  |
| **Female** |  |  |  |  | |  | |  | |  | |  | |  | |  | |  |
| **Stomach** | 4.2 | 3.5 | 3.0 | 2.5 | | 2.1 | | 1.5 | | 1.5 | | 1.4 | | 1.2 | | 1.0 | | 0.6 |
| **Colon** | 7.5 | 6.3 | 5.4 | 4.6 | | 3.9 | | 2.8 | | 2.7 | | 2.6 | | 2.3 | | 1.8 | | 1.1 |
| **Liver** | 1.8 | 1.5 | 1.3 | 1.0 | | 0.9 | | 0.7 | | 0.6 | | 0.6 | | 0.5 | | 0.4 | | 0.2 |
| **Lung** | 47.3 | 39.3 | 32.5 | 27.0 | | 22.4 | | 15.7 | | 15.6 | | 15.0 | | 13.5 | | 10.3 | | 6.0 |
| **Breast** | 20.2 | 15.7 | 12.3 | 9.6 | | 7.4 | | 4.5 | | 2.6 | | 1.4 | | 0.7 | | 0.4 | | 0.1 |
| **Uterus** | 0.8 | 0.7 | 0.6 | 0.5 | | 0.4 | | 0.3 | | 0.3 | | 0.2 | | 0.2 | | 0.1 | | 0.1 |
| **Ovary** | 4.0 | 3.5 | 2.9 | 2.5 | | 2.1 | | 1.5 | | 1.5 | | 1.3 | | 1.1 | | 0.7 | | 0.4 |
| **Bladder** | 4.3 | 3.8 | 3.2 | 2.6 | | 2.3 | | 1.7 | | 1.7 | | 1.6 | | 1.6 | | 1.4 | | 1.0 |
| **Other** | 36.1 | 21.1 | 16.2 | 13.2 | | 10.8 | | 7.6 | | 7.1 | | 6.3 | | 5.1 | | 3.5 | | 1.8 |
| **All solids** | 126.3 | 95.3 | 77.3 | 63.4 | | 52.3 | | 36.1 | | 33.5 | | 30.5 | | 26.0 | | 19.5 | | 11.2 |
| **Leukemia** | 3.9 | 3.8 | 3.9 | 3.8 | | 3.8 | | 3.8 | | 3.8 | | 4.0 | | 4.0 | | 3.8 | | 2.8 |
| **All cancers** | 130.2 | 99.1 | 81.2 | 67.2 | | 56.1 | | 39.9 | | 37.3 | | 34.5 | | 30.1 | | 23.3 | | 14.0 |

**The values of lifetime risk of various cancers (in 100000 people) induced by annual BRs in Dogonbadan city**

| **Age at exposure time (year)** | | | | | | | | | | | | |
| --- | --- | --- | --- | --- | --- | --- | --- | --- | --- | --- | --- | --- |
|  | **0** | **5** | **10** | **15** | **20** | **30** | **40** | **50** | **60** | **70** | **80** |  |
| **Male** |  |  |  |  |  |  |  |  |  |  |  |  |
| **Stomach** | 4.8 | 4.1 | 3.5 | 2.9 | 2.5 | 1.8 | 1.7 | 1.6 | 1.3 | 0.9 | 0.4 |  |
| **Colon** | 21.4 | 18.1 | 15.3 | 13.0 | 11.0 | 7.9 | 7.8 | 7.2 | 6.0 | 4.1 | 1.9 |  |
| **Liver** | 3.9 | 3.2 | 2.7 | 2.3 | 1.9 | 1.4 | 1.3 | 1.2 | 0.9 | 0.5 | 0.2 |  |
| **Lung** | 20.0 | 16.6 | 13.7 | 11.4 | 9.5 | 6.7 | 6.6 | 6.4 | 5.7 | 4.1 | 2.2 |  |
| **Prostate** | 5.9 | 5.1 | 4.3 | 3.6 | 3.1 | 2.2 | 2.2 | 2.1 | 1.7 | 0.9 | 0.3 |  |
| **Bladder** | 13.3 | 11.3 | 9.5 | 8.1 | 6.9 | 5.0 | 5.0 | 4.8 | 4.2 | 3.0 | 1.5 |  |
| **Other** | 71.4 | 42.7 | 32.0 | 25.0 | 19.8 | 12.6 | 10.9 | 8.9 | 6.2 | 3.6 | 1.5 |  |
| **Thyroid** | 7.3 | 4.8 | 3.2 | 2.1 | 1.3 | 0.6 | 0.2 | 0.1 | 0.0 | 0.0 | 0.0 |  |
| **All solids** | 147.9 | 106.0 | 84.2 | 68.4 | 56.0 | 38.3 | 35.9 | 32.2 | 25.9 | 17.2 | 8.0 |  |
| **Leukemia** | 15.1 | 9.5 | 7.6 | 6.7 | 6.1 | 5.3 | 5.3 | 5.3 | 5.2 | 4.6 | 3.1 |  |
| **All cancers** | 162.9 | 115.4 | 91.9 | 75.1 | 62.1 | 43.6 | 41.2 | 37.6 | 31.1 | 21.8 | 11.1 |  |
|  |  |  |  |  |  |  |  |  |  |  |  |  |
| **Female** |  |  |  |  |  |  |  |  |  |  |  |  |
| **Stomach** | 6.4 | 5.4 | 4.6 | 3.9 | 3.3 | 2.3 | 2.2 | 2.0 | 1.7 | 1.2 | 0.7 |  |
| **Colon** | 14.0 | 11.9 | 10.0 | 8.5 | 7.2 | 5.2 | 5.0 | 4.6 | 3.9 | 2.9 | 1.5 |  |
| **Liver** | 1.8 | 1.5 | 1.3 | 1.0 | 0.9 | 0.6 | 0.6 | 0.6 | 0.4 | 0.3 | 0.1 |  |
| **Lung** | 46.6 | 38.7 | 32.0 | 26.5 | 22.0 | 15.4 | 15.3 | 14.6 | 12.8 | 9.3 | 4.9 |  |
| **Breast** | 74.4 | 58.1 | 45.3 | 35.2 | 27.3 | 16.1 | 9.0 | 4.5 | 2.0 | 0.8 | 0.3 |  |
| **Uterus** | 3.2 | 2.7 | 2.3 | 1.9 | 1.7 | 1.1 | 1.0 | 0.8 | 0.6 | 0.3 | 0.1 |  |
| **Ovary** | 6.6 | 90.5 | 4.6 | 3.8 | 3.2 | 2.2 | 2.0 | 1.6 | 1.1 | 0.7 | 0.3 |  |
| **Bladder** | 13.5 | 11.4 | 9.7 | 8.2 | 6.9 | 5.0 | 5.0 | 4.7 | 4.1 | 3.0 | 1.5 |  |
| **Other** | 85.1 | 45.7 | 33.2 | 26.0 | 20.5 | 13.2 | 11.5 | 9.4 | 6.9 | 4.3 | 1.9 |  |
| **Thyroid** | 40.3 | 26.6 | 17.5 | 11.3 | 7.2 | 2.6 | 0.9 | 0.3 | 0.1 | 0.0 | 0.0 |  |
| **All solids** | 291.9 | 207.6 | 160.5 | 126.4 | 100.1 | 63.7 | 52.4 | 43.1 | 33.6 | 22.8 | 11.3 |  |
| **Leukemia** | 11.8 | 7.1 | 5.5 | 4.8 | 4.5 | 4.0 | 3.9 | 3.9 | 3.6 | 3.2 | 2.4 |  |
| **All cancers** | 303.7 | 214.7 | 166.0 | 131.2 | 104.6 | 67.7 | 56.3 | 47.0 | 37.3 | 26.0 | 13.6 |  |

**The values of lifetime risk of various cancers mortalities (in 100000 people) induced by annual BRs in Dogonbadan city**

| **Age at exposure time (year)** | | | | | | | | | | | | | | | | | | |
| --- | --- | --- | --- | --- | --- | --- | --- | --- | --- | --- | --- | --- | --- | --- | --- | --- | --- | --- |
|  | **0** | **5** | **10** | **15** | **20** | | **30** | | **40** | | **50** | | **60** | | **70** | | **80** | |
| **Male** |  |  |  |  |  | |  | |  | |  | |  | |  | |  | |
| **Stomach** | 2.6 | 2.2 | 1.9 | 1.6 | | 1.3 | | 1.0 | | 1.0 | | 0.8 | | 0.7 | | 0.5 | | 0.3 |
| **Colon** | 10.4 | 8.8 | 7.4 | 6.3 | | 5.3 | | 3.9 | | 3.8 | | 3.6 | | 3.1 | | 2.3 | | 1.3 |
| **Liver** | 2.8 | 2.4 | 2.0 | 1.7 | | 1.5 | | 1.0 | | 1.0 | | 0.9 | | 0.8 | | 0.5 | | 0.3 |
| **Lung** | 20.2 | 16.8 | 13.9 | 11.6 | | 9.6 | | 6.8 | | 6.8 | | 6.6 | | 5.9 | | 4.5 | | 2.7 |
| **Prostate** | 1.1 | 1.0 | 0.8 | 0.6 | | 0.6 | | 0.4 | | 0.4 | | 0.4 | | 0.4 | | 0.4 | | 0.3 |
| **Bladder** | 2.9 | 2.4 | 2.0 | 1.7 | | 1.5 | | 1.1 | | 1.1 | | 1.1 | | 1.1 | | 1.0 | | 0.6 |
| **Other** | 25.4 | 16.2 | 12.7 | 10.3 | | 8.5 | | 6.0 | | 5.6 | | 4.9 | | 3.7 | | 2.3 | | 1.1 |
| **All solids** | 65.4 | 49.6 | 40.7 | 33.9 | | 28.2 | | 20.2 | | 19.7 | | 18.4 | | 15.6 | | 11.5 | | 6.5 |
| **Leukemia** | 4.5 | 4.5 | 4.5 | 4.5 | | 4.3 | | 4.1 | | 4.3 | | 4.5 | | 4.6 | | 4.4 | | 3.2 |
| **All cancers** | 69.9 | 54.2 | 45.3 | 38.3 | | 32.5 | | 24.2 | | 24.0 | | 22.9 | | 20.3 | | 15.9 | | 9.7 |
|  |  |  |  |  | |  | |  | |  | |  | |  | |  | |  |
| **Female** |  |  |  |  | |  | |  | |  | |  | |  | |  | |  |
| **Stomach** | 3.6 | 3.1 | 2.6 | 2.2 | | 1.8 | | 1.3 | | 1.3 | | 1.2 | | 1.0 | | 0.8 | | 0.5 |
| **Colon** | 6.5 | 5.5 | 4.6 | 3.9 | | 3.4 | | 2.4 | | 2.4 | | 2.2 | | 2.0 | | 1.6 | | 1.0 |
| **Liver** | 1.5 | 1.3 | 1.1 | 0.9 | | 0.8 | | 0.6 | | 0.5 | | 0.5 | | 0.4 | | 0.3 | | 0.2 |
| **Lung** | 40.9 | 33.9 | 28.1 | 23.3 | | 19.4 | | 13.5 | | 13.5 | | 13.0 | | 11.6 | | 8.9 | | 5.1 |
| **Breast** | 17.4 | 13.6 | 10.6 | 8.3 | | 6.4 | | 3.9 | | 2.2 | | 1.2 | | 0.6 | | 0.3 | | 0.1 |
| **Uterus** | 0.7 | 0.6 | 0.5 | 0.4 | | 0.4 | | 0.3 | | 0.3 | | 0.2 | | 0.2 | | 0.1 | | 0.1 |
| **Ovary** | 3.5 | 48.9 | 2.5 | 2.2 | | 1.8 | | 1.3 | | 1.3 | | 1.1 | | 1.0 | | 0.6 | | 0.3 |
| **Bladder** | 3.8 | 3.2 | 2.7 | 2.3 | | 2.0 | | 1.5 | | 1.5 | | 1.4 | | 1.4 | | 1.2 | | 0.8 |
| **Other** | 31.2 | 18.2 | 14.0 | 11.4 | | 9.3 | | 6.5 | | 6.2 | | 5.5 | | 4.4 | | 3.0 | | 1.5 |
| **All solids** | 109.2 | 82.3 | 66.8 | 54.8 | | 45.2 | | 31.2 | | 28.9 | | 26.4 | | 22.5 | | 16.8 | | 9.7 |
| **Leukemia** | 3.4 | 3.3 | 3.4 | 3.3 | | 3.2 | | 3.2 | | 3.3 | | 3.4 | | 3.5 | | 3.3 | | 2.4 |
| **All cancers** | 112.5 | 85.6 | 70.2 | 58.1 | | 48.4 | | 34.5 | | 32.2 | | 29.8 | | 26.0 | | 20.2 | | 12.1 |

**The values of lifetime risk of various cancers (in 100000 people) induced by annual BRs in Dehdasht city**

| **Age at exposure time (year)** | | | | | | | | | | | |
| --- | --- | --- | --- | --- | --- | --- | --- | --- | --- | --- | --- |
|  | **0** | **5** | **10** | **15** | **20** | **30** | **40** | **50** | **60** | **70** | **80** |
| **Male** |  |  |  |  |  |  |  |  |  |  |  |
| **Stomach** | 5.0 | 4.3 | 3.7 | 3.1 | 2.7 | 1.9 | 1.8 | 1.7 | 1.3 | 0.9 | 0.5 |
| **Colon** | 22.3 | 18.9 | 16.0 | 13.6 | 11.5 | 8.3 | 8.1 | 7.5 | 6.2 | 4.3 | 2.0 |
| **Liver** | 4.1 | 3.3 | 2.9 | 2.4 | 2.0 | 1.5 | 1.4 | 1.3 | 0.9 | 0.5 | 0.2 |
| **Lung** | 20.9 | 17.3 | 14.3 | 12.0 | 9.9 | 7.0 | 6.9 | 6.7 | 5.9 | 4.3 | 2.3 |
| **Prostate** | 6.2 | 5.3 | 4.5 | 3.8 | 3.2 | 2.3 | 2.3 | 2.2 | 1.7 | 0.9 | 0.3 |
| **Bladder** | 13.9 | 11.8 | 10.0 | 8.4 | 7.2 | 5.2 | 5.2 | 5.0 | 4.4 | 3.1 | 1.5 |
| **Other** | 74.6 | 44.6 | 33.4 | 26.2 | 20.7 | 13.2 | 11.4 | 9.3 | 6.5 | 3.8 | 1.5 |
| **Thyroid** | 7.6 | 5.0 | 3.3 | 2.2 | 1.4 | 0.6 | 0.2 | 0.1 | 0.0 | 0.0 | 0.0 |
| **All solids** | 154.5 | 110.7 | 88.0 | 71.5 | 58.5 | 40.0 | 37.5 | 33.7 | 27.0 | 17.9 | 8.4 |
| **Leukemia** | 15.7 | 9.9 | 8.0 | 7.0 | 6.4 | 5.6 | 5.6 | 5.6 | 5.4 | 4.8 | 3.2 |
| **All cancers** | 170.3 | 120.6 | 96.0 | 78.5 | 64.9 | 45.6 | 43.0 | 39.3 | 32.5 | 22.8 | 11.6 |
|  |  |  |  |  |  |  |  |  |  |  |  |
| **Female** |  |  |  |  |  |  |  |  |  |  |  |
| **Stomach** | 6.7 | 5.6 | 4.8 | 4.1 | 3.5 | 2.4 | 2.3 | 2.1 | 1.8 | 1.3 | 0.7 |
| **Colon** | 14.6 | 12.4 | 10.5 | 8.9 | 7.6 | 5.4 | 5.2 | 4.8 | 4.1 | 3.0 | 1.5 |
| **Liver** | 1.9 | 1.5 | 1.3 | 1.1 | 0.9 | 0.7 | 0.7 | 0.6 | 0.5 | 0.3 | 0.1 |
| **Lung** | 48.7 | 40.4 | 33.5 | 27.7 | 23.0 | 16.1 | 15.9 | 15.3 | 13.4 | 9.8 | 5.1 |
| **Breast** | 77.8 | 60.7 | 47.3 | 36.7 | 28.5 | 16.8 | 9.4 | 4.7 | 2.1 | 0.8 | 0.3 |
| **Uterus** | 3.3 | 2.8 | 2.4 | 2.0 | 1.7 | 1.2 | 1.1 | 0.9 | 0.6 | 0.3 | 0.1 |
| **Ovary** | 6.9 | 90.5 | 4.8 | 4.0 | 3.3 | 2.3 | 2.1 | 1.7 | 1.2 | 0.7 | 0.3 |
| **Bladder** | 14.1 | 12.0 | 10.1 | 8.6 | 7.2 | 5.2 | 5.2 | 4.9 | 4.3 | 3.1 | 1.6 |
| **Other** | 88.9 | 47.8 | 34.7 | 27.2 | 21.5 | 13.8 | 12.0 | 9.8 | 7.2 | 4.5 | 2.0 |
| **Thyroid** | 42.1 | 27.8 | 18.3 | 11.8 | 7.5 | 2.7 | 0.9 | 0.3 | 0.1 | 0.0 | 0.0 |
| **All solids** | 305.0 | 216.9 | 167.7 | 132.1 | 104.6 | 66.6 | 54.7 | 45.0 | 35.1 | 23.8 | 11.8 |
| **Leukemia** | 12.3 | 7.4 | 5.7 | 5.0 | 4.7 | 4.2 | 4.1 | 4.1 | 3.8 | 3.4 | 2.5 |
| **All cancers** | 317.3 | 224.3 | 173.4 | 137.1 | 109.3 | 70.7 | 58.9 | 49.2 | 38.9 | 27.2 | 14.2 |

**The values of lifetime risk of various cancers mortalities (in 100000 people) induced by annual BRs in Dehdasht city**

| **Age at exposure time (year)** | | | | | | | | | | | | | | | | | | | |
| --- | --- | --- | --- | --- | --- | --- | --- | --- | --- | --- | --- | --- | --- | --- | --- | --- | --- | --- | --- |
|  | **0** | **5** | **10** | **15** | **20** | | **30** | | **40** | | **50** | | **60** | | **70** | | **80** | |  |
| **Male** |  |  |  |  |  | |  | |  | |  | |  | |  | |  | |  |
| **Stomach** | 2.7 | 2.3 | 2.0 | 1.7 | | 1.4 | | 1.1 | | 1.0 | | 0.9 | | 0.7 | | 0.5 | | 0.3 |  |
| **Colon** | 10.8 | 9.2 | 7.8 | 6.6 | | 5.6 | | 4.1 | | 4.0 | | 3.8 | | 3.3 | | 2.4 | | 1.4 |  |
| **Liver** | 2.9 | 2.5 | 2.1 | 1.8 | | 1.5 | | 1.1 | | 1.1 | | 0.9 | | 0.8 | | 0.5 | | 0.3 |  |
| **Lung** | 21.1 | 17.5 | 14.5 | 12.1 | | 10.0 | | 7.1 | | 7.1 | | 6.9 | | 6.2 | | 4.7 | | 2.8 |  |
| **Prostate** | 1.1 | 1.0 | 0.8 | 0.7 | | 0.6 | | 0.5 | | 0.4 | | 0.5 | | 0.5 | | 0.5 | | 0.3 |  |
| **Bladder** | 3.0 | 2.5 | 2.1 | 1.8 | | 1.5 | | 1.1 | | 1.1 | | 1.1 | | 1.1 | | 1.0 | | 0.7 |  |
| **Other** | 26.6 | 16.9 | 13.3 | 10.8 | | 8.9 | | 6.2 | | 5.8 | | 5.1 | | 3.9 | | 2.4 | | 1.1 |  |
| **All solids** | 68.3 | 51.9 | 42.6 | 35.4 | | 29.5 | | 21.1 | | 20.6 | | 19.2 | | 16.3 | | 12.0 | | 6.8 |  |
| **Leukemia** | 4.7 | 4.7 | 4.7 | 4.7 | | 4.5 | | 4.3 | | 4.5 | | 4.7 | | 4.8 | | 4.6 | | 3.4 |  |
| **All cancers** | 73.0 | 56.6 | 47.3 | 40.1 | | 33.9 | | 25.3 | | 25.0 | | 23.9 | | 21.2 | | 16.6 | | 10.2 |  |
|  |  |  |  |  | |  | |  | |  | |  | |  | |  | |  |  |
| **Female** |  |  |  |  | |  | |  | |  | |  | |  | |  | |  |  |
| **Stomach** | 3.8 | 3.2 | 2.7 | 2.3 | | 1.9 | | 1.4 | | 1.3 | | 1.3 | | 1.1 | | 0.9 | | 0.5 |  |
| **Colon** | 6.8 | 5.7 | 4.8 | 4.1 | | 3.5 | | 2.5 | | 2.5 | | 2.3 | | 2.1 | | 1.7 | | 1.0 |  |
| **Liver** | 1.6 | 1.3 | 1.1 | 0.9 | | 0.8 | | 0.6 | | 0.5 | | 0.5 | | 0.5 | | 0.3 | | 0.2 |  |
| **Lung** | 42.7 | 35.5 | 29.4 | 24.4 | | 20.3 | | 14.1 | | 14.1 | | 13.6 | | 12.2 | | 9.3 | | 5.4 |  |
| **Breast** | 18.2 | 14.2 | 11.1 | 8.6 | | 6.7 | | 4.1 | | 2.3 | | 1.3 | | 0.6 | | 0.3 | | 0.1 |  |
| **Uterus** | 0.7 | 0.7 | 0.5 | 0.5 | | 0.4 | | 0.3 | | 0.3 | | 0.2 | | 0.2 | | 0.1 | | 0.1 |  |
| **Ovary** | 3.7 | 48.9 | 2.6 | 2.3 | | 1.9 | | 1.3 | | 1.3 | | 1.2 | | 1.0 | | 0.7 | | 0.3 |  |
| **Bladder** | 3.9 | 3.4 | 2.9 | 2.4 | | 2.1 | | 1.5 | | 1.5 | | 1.5 | | 1.5 | | 1.3 | | 0.9 |  |
| **Other** | 32.6 | 19.1 | 14.6 | 11.9 | | 9.8 | | 6.8 | | 6.4 | | 5.7 | | 4.6 | | 3.1 | | 1.6 |  |
| **All solids** | 114.1 | 86.0 | 69.8 | 57.3 | | 47.2 | | 32.6 | | 30.2 | | 27.6 | | 23.5 | | 17.6 | | 10.1 |  |
| **Leukemia** | 3.5 | 3.5 | 3.5 | 3.5 | | 3.4 | | 3.4 | | 3.5 | | 3.6 | | 3.7 | | 3.5 | | 2.5 |  |
| **All cancers** | 117.6 | 89.5 | 73.3 | 60.7 | | 50.6 | | 36.0 | | 33.7 | | 31.2 | | 27.2 | | 21.1 | | 12.6 |  |

**The values of lifetime risk of various cancers (in 100000 people) induced by annual BRs in Sisakht city**

| **Age at exposure time (year)** | | | | | | | | | | | |
| --- | --- | --- | --- | --- | --- | --- | --- | --- | --- | --- | --- |
|  | **0** | **5** | **10** | **15** | **20** | **30** | **40** | **50** | **60** | **70** | **80** |
| **Male** |  |  |  |  |  |  |  |  |  |  |  |
| **Stomach** | 5.5 | 4.7 | 4.0 | 3.4 | 2.9 | 2.0 | 2.0 | 1.8 | 1.5 | 1.0 | 0.5 |
| **Colon** | 24.5 | 20.8 | 17.6 | 14.9 | 12.6 | 9.1 | 8.9 | 8.2 | 6.8 | 4.7 | 2.2 |
| **Liver** | 4.4 | 3.6 | 3.1 | 2.6 | 2.2 | 1.6 | 1.5 | 1.4 | 1.0 | 0.6 | 0.2 |
| **Lung** | 22.9 | 19.0 | 15.7 | 13.1 | 10.9 | 7.7 | 7.6 | 7.4 | 6.5 | 4.7 | 2.5 |
| **Prostate** | 6.8 | 5.8 | 4.9 | 4.2 | 3.5 | 2.6 | 2.6 | 2.4 | 1.9 | 1.0 | 0.4 |
| **Bladder** | 15.2 | 12.9 | 10.9 | 9.3 | 7.9 | 5.8 | 5.8 | 5.5 | 4.8 | 3.4 | 1.7 |
| **Other** | 81.8 | 49.0 | 36.6 | 28.7 | 22.7 | 14.4 | 12.5 | 10.2 | 7.1 | 4.2 | 1.7 |
| **Thyroid** | 8.4 | 5.5 | 3.6 | 2.4 | 1.5 | 0.7 | 0.2 | 0.1 | 0.0 | 0.0 | 0.0 |
| **All solids** | 169.5 | 121.5 | 96.5 | 78.4 | 64.2 | 43.9 | 41.1 | 36.9 | 29.7 | 19.7 | 9.2 |
| **Leukemia** | 17.3 | 10.9 | 8.7 | 7.7 | 7.0 | 6.1 | 6.1 | 6.1 | 6.0 | 5.3 | 3.5 |
| **All cancers** | 186.7 | 132.3 | 105.3 | 86.1 | 71.2 | 50.0 | 47.2 | 43.1 | 35.6 | 25.0 | 12.7 |
|  |  |  |  |  |  |  |  |  |  |  |  |
| **Female** |  |  |  |  |  |  |  |  |  |  |  |
| **Stomach** | 7.4 | 6.2 | 5.2 | 4.4 | 3.8 | 2.6 | 2.6 | 2.3 | 2.0 | 1.4 | 0.8 |
| **Colon** | 16.0 | 13.6 | 11.5 | 9.8 | 8.3 | 6.0 | 5.8 | 5.3 | 4.5 | 3.3 | 1.7 |
| **Liver** | 2.0 | 1.7 | 1.5 | 1.2 | 1.0 | 0.7 | 0.7 | 0.7 | 0.5 | 0.4 | 0.1 |
| **Lung** | 53.4 | 44.3 | 36.7 | 30.4 | 25.2 | 17.6 | 17.5 | 16.8 | 14.6 | 10.7 | 5.6 |
| **Breast** | 85.3 | 66.6 | 51.9 | 40.3 | 31.3 | 18.4 | 10.3 | 5.1 | 2.3 | 0.9 | 0.3 |
| **Uterus** | 3.6 | 3.1 | 2.6 | 2.2 | 1.9 | 1.3 | 1.2 | 0.9 | 0.7 | 0.4 | 0.1 |
| **Ovary** | 7.6 | 90.5 | 5.3 | 4.4 | 3.6 | 2.5 | 2.3 | 1.8 | 1.3 | 0.8 | 0.4 |
| **Bladder** | 15.4 | 13.1 | 11.1 | 9.4 | 7.9 | 5.8 | 5.7 | 5.4 | 4.7 | 3.4 | 1.7 |
| **Other** | 97.6 | 52.4 | 38.1 | 29.8 | 23.5 | 15.1 | 13.2 | 10.8 | 7.9 | 5.0 | 2.2 |
| **Thyroid** | 46.2 | 30.5 | 20.0 | 13.0 | 8.2 | 3.0 | 1.0 | 0.3 | 0.1 | 0.0 | 0.0 |
| **All solids** | 334.6 | 237.9 | 184.0 | 144.8 | 114.8 | 73.0 | 60.0 | 49.4 | 38.5 | 26.1 | 12.9 |
| **Leukemia** | 13.5 | 8.2 | 6.3 | 5.5 | 5.2 | 4.6 | 4.5 | 4.5 | 4.2 | 3.7 | 2.7 |
| **All cancers** | 348.0 | 246.0 | 190.2 | 150.4 | 119.9 | 77.6 | 64.6 | 53.9 | 42.7 | 29.8 | 15.6 |

**The values of lifetime risk of various cancers mortalities (in 100000 people) induced by annual BRs in Sisakht city**

| **Age at exposure time (year)** | | | | | | | | | | | | | | | | |
| --- | --- | --- | --- | --- | --- | --- | --- | --- | --- | --- | --- | --- | --- | --- | --- | --- |
|  | **0** | **5** | **10** | **15** | **20** | | **30** | **40** | | **50** | **60** | | | **70** | **80** | |
| **Male** |  |  |  |  |  | |  |  | |  |  | | |  |  | |
| **Stomach** | 3.0 | 2.5 | 2.2 | 1.8 | 1.5 | 1.2 | | 1.1 | 0.9 | | | 0.8 | 0.6 | | | 0.3 |
| **Colon** | 11.9 | 10.1 | 8.5 | 7.2 | 6.1 | 4.4 | | 4.4 | 4.2 | | | 3.6 | 2.6 | | | 1.5 |
| **Liver** | 3.2 | 2.7 | 2.3 | 2.0 | 1.7 | 1.2 | | 1.2 | 1.0 | | | 0.9 | 0.6 | | | 0.3 |
| **Lung** | 23.2 | 19.2 | 16.0 | 13.3 | 11.0 | 7.8 | | 7.8 | 7.6 | | | 6.8 | 5.2 | | | 3.1 |
| **Prostate** | 1.2 | 1.1 | 0.9 | 0.7 | 0.7 | 0.5 | | 0.4 | 0.5 | | | 0.5 | 0.5 | | | 0.4 |
| **Bladder** | 3.3 | 2.8 | 2.3 | 2.0 | 1.7 | 1.2 | | 1.2 | 1.2 | | | 1.2 | 1.1 | | | 0.7 |
| **Other** | 29.1 | 18.6 | 14.6 | 11.8 | 9.8 | 6.8 | | 6.4 | 5.6 | | | 4.2 | 2.6 | | | 1.2 |
| **All solids** | 74.9 | 56.9 | 46.7 | 38.8 | 32.3 | 23.1 | | 22.6 | 21.1 | | | 17.9 | 13.2 | | | 7.4 |
| **Leukemia** | 5.2 | 5.2 | 5.2 | 5.1 | 4.9 | 4.7 | | 4.9 | 5.2 | | | 5.3 | 5.0 | | | 3.7 |
| **All cancers** | 80.1 | 62.1 | 51.9 | 43.9 | 37.2 | 27.8 | | 27.5 | 26.2 | | | 23.2 | 18.2 | | | 11.1 |
|  |  |  |  |  |  |  | |  |  | | |  |  | | |  |
| **Female** |  |  |  |  |  |  | |  |  | | |  |  | | |  |
| **Stomach** | 4.2 | 3.5 | 3.0 | 2.5 | 2.1 | 1.5 | | 1.5 | 1.4 | | | 1.2 | 0.9 | | | 0.6 |
| **Colon** | 7.4 | 6.3 | 5.3 | 4.5 | 3.9 | 2.8 | | 2.7 | 2.6 | | | 2.3 | 1.8 | | | 1.1 |
| **Liver** | 1.7 | 1.5 | 1.2 | 1.0 | 0.9 | 0.7 | | 0.6 | 0.6 | | | 0.5 | 0.4 | | | 0.2 |
| **Lung** | 46.8 | 38.9 | 32.2 | 26.7 | 22.2 | 15.5 | | 15.4 | 14.9 | | | 13.3 | 10.2 | | | 5.9 |
| **Breast** | 20.0 | 15.6 | 12.2 | 9.5 | 7.4 | 4.4 | | 2.6 | 1.4 | | | 0.7 | 0.4 | | | 0.1 |
| **Uterus** | 0.8 | 0.7 | 0.6 | 0.5 | 0.4 | 0.3 | | 0.3 | 0.2 | | | 0.2 | 0.1 | | | 0.1 |
| **Ovary** | 4.0 | 48.9 | 2.8 | 2.5 | 2.0 | 1.5 | | 1.5 | 1.3 | | | 1.1 | 0.7 | | | 0.4 |
| **Bladder** | 4.3 | 3.7 | 3.1 | 2.6 | 2.3 | 1.7 | | 1.7 | 1.6 | | | 1.6 | 1.4 | | | 0.9 |
| **Other** | 35.8 | 20.9 | 16.0 | 13.0 | 10.7 | 7.5 | | 7.1 | 6.3 | | | 5.0 | 3.4 | | | 1.7 |
| **All solids** | 125.1 | 94.4 | 76.6 | 62.8 | 51.8 | 35.8 | | 33.2 | 30.2 | | | 25.8 | 19.3 | | | 11.1 |
| **Leukemia** | 3.9 | 3.8 | 3.9 | 3.8 | 3.7 | 3.7 | | 3.8 | 3.9 | | | 4.0 | 3.8 | | | 2.8 |
| **All cancers** | 129.0 | 98.1 | 80.4 | 66.6 | 55.5 | 39.5 | | 36.9 | 34.2 | | | 29.8 | 23.1 | | | 13.8 |

**The values of lifetime risk of various cancers (in 100000 people) induced by annual BRs in Basht city**

| **Age at exposure time (year)** | | | | | | | | | | | |
| --- | --- | --- | --- | --- | --- | --- | --- | --- | --- | --- | --- |
|  | **0** | **5** | **10** | **15** | **20** | **30** | **40** | **50** | **60** | **70** | **80** |
| **Male** |  |  |  |  |  |  |  |  |  |  |  |
| **Stomach** | 4.8 | 4.1 | 3.5 | 2.9 | 2.5 | 1.8 | 1.7 | 1.6 | 1.3 | 0.9 | 0.4 |
| **Colon** | 21.1 | 17.9 | 15.1 | 12.8 | 10.9 | 7.9 | 7.7 | 7.1 | 5.9 | 4.1 | 1.9 |
| **Liver** | 3.8 | 3.1 | 2.7 | 2.3 | 1.9 | 1.4 | 1.3 | 1.2 | 0.9 | 0.5 | 0.2 |
| **Lung** | 19.7 | 16.4 | 13.6 | 11.3 | 9.4 | 6.6 | 6.5 | 6.3 | 5.6 | 4.1 | 2.1 |
| **Prostate** | 5.8 | 5.0 | 4.2 | 3.6 | 3.0 | 2.2 | 2.2 | 2.1 | 1.6 | 0.9 | 0.3 |
| **Bladder** | 13.1 | 11.1 | 9.4 | 8.0 | 6.8 | 5.0 | 5.0 | 4.8 | 4.1 | 3.0 | 1.4 |
| **Other** | 70.6 | 42.2 | 31.6 | 24.8 | 19.6 | 12.4 | 10.8 | 8.8 | 6.2 | 3.6 | 1.4 |
| **Thyroid** | 7.2 | 4.8 | 3.1 | 2.1 | 1.3 | 0.6 | 0.2 | 0.1 | 0.0 | 0.0 | 0.0 |
| **All solids** | 146.2 | 104.8 | 83.3 | 67.6 | 55.4 | 37.8 | 35.5 | 31.9 | 25.6 | 17.0 | 7.9 |
| **Leukemia** | 14.9 | 9.4 | 7.5 | 6.6 | 6.0 | 5.3 | 5.3 | 5.3 | 5.2 | 4.6 | 3.0 |
| **All cancers** | 161.1 | 114.1 | 90.8 | 74.3 | 61.4 | 43.1 | 40.7 | 37.1 | 30.7 | 21.6 | 10.9 |
|  |  |  |  |  |  |  |  |  |  |  |  |
| **Female** |  |  |  |  |  |  |  |  |  |  |  |
| **Stomach** | 6.3 | 5.3 | 4.5 | 3.8 | 3.3 | 2.3 | 2.2 | 2.0 | 1.7 | 1.2 | 0.7 |
| **Colon** | 13.8 | 11.8 | 9.9 | 8.4 | 7.2 | 5.2 | 5.0 | 4.6 | 3.9 | 2.8 | 1.4 |
| **Liver** | 1.8 | 1.4 | 1.3 | 1.0 | 0.9 | 0.6 | 0.6 | 0.6 | 0.4 | 0.3 | 0.1 |
| **Lung** | 46.1 | 38.2 | 31.7 | 26.2 | 21.7 | 15.2 | 15.1 | 14.5 | 12.6 | 9.2 | 4.8 |
| **Breast** | 73.6 | 57.5 | 44.8 | 34.8 | 27.0 | 15.9 | 8.9 | 4.4 | 1.9 | 0.8 | 0.3 |
| **Uterus** | 3.1 | 2.6 | 2.3 | 1.9 | 1.6 | 1.1 | 1.0 | 0.8 | 0.6 | 0.3 | 0.1 |
| **Ovary** | 6.5 | 90.5 | 4.6 | 3.8 | 3.1 | 2.1 | 1.9 | 1.6 | 1.1 | 0.7 | 0.3 |
| **Bladder** | 13.3 | 11.3 | 9.6 | 8.1 | 6.9 | 5.0 | 4.9 | 4.7 | 4.0 | 3.0 | 1.5 |
| **Other** | 84.2 | 45.2 | 32.9 | 25.7 | 20.3 | 13.0 | 11.4 | 9.3 | 6.9 | 4.3 | 1.9 |
| **Thyroid** | 39.9 | 26.3 | 17.3 | 11.2 | 7.1 | 2.6 | 0.9 | 0.3 | 0.1 | 0.0 | 0.0 |
| **All solids** | 288.6 | 205.2 | 158.7 | 125.0 | 99.0 | 63.0 | 51.8 | 42.6 | 33.3 | 22.5 | 11.1 |
| **Leukemia** | 11.6 | 7.0 | 5.4 | 4.8 | 4.5 | 4.0 | 3.9 | 3.9 | 3.6 | 3.2 | 2.3 |
| **All cancers** | 300.3 | 212.3 | 164.1 | 129.7 | 103.5 | 66.9 | 55.7 | 46.5 | 36.8 | 25.7 | 13.5 |

**The values of lifetime risk of various cancers mortalities (in 100000 people) induced by annual BRs in Basht city**

| **Age at exposure time (year)** | | | | | | | | | | | | | | | | |
| --- | --- | --- | --- | --- | --- | --- | --- | --- | --- | --- | --- | --- | --- | --- | --- | --- |
|  | **0** | **5** | **10** | **15** | **20** | | **30** | **40** | | **50** | **60** | | | **70** | **80** | |
| **Male** |  |  |  |  |  | |  |  | |  |  | | |  |  | |
| **Stomach** | 2.6 | 2.1 | 1.9 | 1.6 | 1.3 | 1.0 | | 0.9 | 0.8 | | | 0.7 | 0.5 | | | 0.3 |
| **Colon** | 10.2 | 8.7 | 7.4 | 6.2 | 5.3 | 3.8 | | 3.8 | 3.6 | | | 3.1 | 2.3 | | | 1.3 |
| **Liver** | 2.8 | 2.3 | 1.9 | 1.7 | 1.4 | 1.0 | | 1.0 | 0.9 | | | 0.8 | 0.5 | | | 0.3 |
| **Lung** | 20.0 | 16.6 | 13.8 | 11.4 | 9.5 | 6.7 | | 6.7 | 6.5 | | | 5.8 | 4.5 | | | 2.6 |
| **Prostate** | 1.1 | 0.9 | 0.8 | 0.6 | 0.6 | 0.4 | | 0.4 | 0.4 | | | 0.4 | 0.4 | | | 0.3 |
| **Bladder** | 2.8 | 2.4 | 2.0 | 1.7 | 1.4 | 1.1 | | 1.1 | 1.1 | | | 1.1 | 0.9 | | | 0.6 |
| **Other** | 25.1 | 16.0 | 12.6 | 10.2 | 8.4 | 5.9 | | 5.5 | 4.8 | | | 3.6 | 2.3 | | | 1.1 |
| **All solids** | 64.6 | 49.1 | 40.3 | 33.5 | 27.9 | 19.9 | | 19.5 | 18.2 | | | 15.5 | 11.4 | | | 6.4 |
| **Leukemia** | 4.5 | 4.5 | 4.5 | 4.4 | 4.2 | 4.0 | | 4.2 | 4.5 | | | 4.6 | 4.3 | | | 3.2 |
| **All cancers** | 69.1 | 53.6 | 44.8 | 37.9 | 32.1 | 23.9 | | 23.7 | 22.6 | | | 20.1 | 15.7 | | | 9.6 |
|  |  |  |  |  |  |  | |  |  | | |  |  | | |  |
| **Female** |  |  |  |  |  |  | |  |  | | |  |  | | |  |
| **Stomach** | 3.6 | 3.0 | 2.6 | 2.1 | 1.8 | 1.3 | | 1.3 | 1.2 | | | 1.0 | 0.8 | | | 0.5 |
| **Colon** | 6.4 | 5.4 | 4.6 | 3.9 | 3.3 | 2.4 | | 2.3 | 2.2 | | | 1.9 | 1.6 | | | 0.9 |
| **Liver** | 1.5 | 1.3 | 1.1 | 0.9 | 0.8 | 0.6 | | 0.5 | 0.5 | | | 0.4 | 0.3 | | | 0.2 |
| **Lung** | 40.4 | 33.6 | 27.8 | 23.1 | 19.2 | 13.4 | | 13.3 | 12.8 | | | 11.5 | 8.8 | | | 5.1 |
| **Breast** | 17.2 | 13.5 | 10.5 | 8.2 | 6.3 | 3.8 | | 2.2 | 1.2 | | | 0.6 | 0.3 | | | 0.1 |
| **Uterus** | 0.7 | 0.6 | 0.5 | 0.4 | 0.4 | 0.3 | | 0.3 | 0.2 | | | 0.2 | 0.1 | | | 0.1 |
| **Ovary** | 3.5 | 48.9 | 2.5 | 2.1 | 1.8 | 1.3 | | 1.3 | 1.1 | | | 0.9 | 0.6 | | | 0.3 |
| **Bladder** | 3.7 | 3.2 | 2.7 | 2.3 | 1.9 | 1.4 | | 1.4 | 1.4 | | | 1.4 | 1.2 | | | 0.8 |
| **Other** | 30.9 | 18.0 | 13.8 | 11.3 | 9.2 | 6.5 | | 6.1 | 5.4 | | | 4.3 | 3.0 | | | 1.5 |
| **All solids** | 107.9 | 81.4 | 66.1 | 54.2 | 44.7 | 30.9 | | 28.6 | 26.1 | | | 22.3 | 16.7 | | | 9.6 |
| **Leukemia** | 3.3 | 3.3 | 3.3 | 3.3 | 3.2 | 3.2 | | 3.3 | 3.4 | | | 3.5 | 3.3 | | | 2.4 |
| **All cancers** | 111.3 | 84.7 | 69.4 | 57.5 | 47.9 | 34.1 | | 31.9 | 29.5 | | | 25.7 | 19.9 | | | 11.9 |

**The values of lifetime risk of various cancers (in 100000 people) induced by annual BRs in Choram city**

| **Age at exposure time (year)** | | | | | | | | | | | | |
| --- | --- | --- | --- | --- | --- | --- | --- | --- | --- | --- | --- | --- |
|  | **0** | **5** | **10** | **15** | **20** | **30** | **40** | **50** | **60** | **70** | **80** |  |
| **Male** |  |  |  |  |  |  |  |  |  |  |  |  |
| **Stomach** | 4.2 | 3.6 | 3.1 | 2.6 | 2.2 | 1.6 | 1.5 | 1.4 | 1.1 | 0.8 | 0.4 |  |
| **Colon** | 18.7 | 15.9 | 13.4 | 11.4 | 9.6 | 7.0 | 6.8 | 6.3 | 5.2 | 3.6 | 1.7 |  |
| **Liver** | 3.4 | 2.8 | 2.4 | 2.0 | 1.7 | 1.2 | 1.2 | 1.1 | 0.8 | 0.4 | 0.2 |  |
| **Lung** | 17.5 | 14.5 | 12.0 | 10.0 | 8.3 | 5.9 | 5.8 | 5.6 | 5.0 | 3.6 | 1.9 |  |
| **Prostate** | 5.2 | 4.5 | 3.7 | 3.2 | 2.7 | 2.0 | 2.0 | 1.8 | 1.4 | 0.8 | 0.3 |  |
| **Bladder** | 11.6 | 9.9 | 8.4 | 7.1 | 6.0 | 4.4 | 4.4 | 4.2 | 3.7 | 2.6 | 1.3 |  |
| **Other** | 62.6 | 37.4 | 28.0 | 22.0 | 17.4 | 11.0 | 9.6 | 7.8 | 5.5 | 3.2 | 1.3 |  |
| **Thyroid** | 6.4 | 4.2 | 2.8 | 1.8 | 1.2 | 0.5 | 0.2 | 0.1 | 0.0 | 0.0 | 0.0 |  |
| **All solids** | 129.6 | 92.9 | 73.8 | 59.9 | 49.1 | 33.5 | 31.4 | 28.2 | 22.7 | 15.0 | 7.0 |  |
| **Leukemia** | 13.2 | 8.3 | 6.7 | 5.9 | 5.3 | 4.7 | 4.7 | 4.7 | 4.6 | 4.1 | 2.7 |  |
| **All cancers** | 142.8 | 101.2 | 80.5 | 65.9 | 54.4 | 38.2 | 36.1 | 32.9 | 27.2 | 19.1 | 9.7 |  |
|  |  |  |  |  |  |  |  |  |  |  |  |  |
| **Female** |  |  |  |  |  |  |  |  |  |  |  |  |
| **Stomach** | 5.6 | 4.7 | 4.0 | 3.4 | 2.9 | 2.0 | 2.0 | 1.8 | 1.5 | 1.1 | 0.6 |  |
| **Colon** | 12.3 | 10.4 | 8.8 | 7.5 | 6.4 | 4.6 | 4.4 | 4.1 | 3.5 | 2.5 | 1.3 |  |
| **Liver** | 1.6 | 1.3 | 1.1 | 0.9 | 0.8 | 0.6 | 0.6 | 0.5 | 0.4 | 0.3 | 0.1 |  |
| **Lung** | 40.8 | 33.9 | 28.1 | 23.2 | 19.3 | 13.5 | 13.4 | 12.8 | 11.2 | 8.2 | 4.3 |  |
| **Breast** | 65.2 | 50.9 | 39.7 | 30.8 | 23.9 | 14.1 | 7.9 | 3.9 | 1.7 | 0.7 | 0.2 |  |
| **Uterus** | 2.8 | 2.3 | 2.0 | 1.7 | 1.4 | 1.0 | 0.9 | 0.7 | 0.5 | 0.3 | 0.1 |  |
| **Ovary** | 5.8 | 90.5 | 4.1 | 3.3 | 2.8 | 1.9 | 1.7 | 1.4 | 1.0 | 0.6 | 0.3 |  |
| **Bladder** | 11.8 | 10.0 | 8.5 | 7.2 | 6.1 | 4.4 | 4.3 | 4.1 | 3.6 | 2.6 | 1.3 |  |
| **Other** | 74.6 | 40.1 | 29.1 | 22.8 | 18.0 | 11.5 | 10.1 | 8.2 | 6.1 | 3.8 | 1.7 |  |
| **Thyroid** | 35.3 | 23.3 | 15.3 | 9.9 | 6.3 | 2.3 | 0.8 | 0.2 | 0.1 | 0.0 | 0.0 |  |
| **All solids** | 255.8 | 181.9 | 140.7 | 110.8 | 87.8 | 55.8 | 45.9 | 37.8 | 29.5 | 19.9 | 9.9 |  |
| **Leukemia** | 10.3 | 6.2 | 4.8 | 4.2 | 4.0 | 3.5 | 3.5 | 3.5 | 3.2 | 2.8 | 2.1 |  |
| **All cancers** | 266.1 | 188.1 | 145.5 | 115.0 | 91.7 | 59.3 | 49.4 | 41.2 | 32.6 | 22.8 | 11.9 |  |

**The values of lifetime risk of various cancers mortalities (in 100000 people) induced by annual BRs in Choram city**

| **Age at exposure time (year)** | | | | | | | | | | | | | | | | |
| --- | --- | --- | --- | --- | --- | --- | --- | --- | --- | --- | --- | --- | --- | --- | --- | --- |
|  | **0** | **5** | **10** | **15** | **20** | | **30** | **40** | | **50** | **60** | | | **70** | **80** | |
| **Male** |  |  |  |  |  | |  |  | |  |  | | |  |  | |
| **Stomach** | 2.3 | 1.9 | 1.7 | 1.4 | 1.2 | 0.9 | | 0.8 | 0.7 | | | 0.6 | 0.4 | | | 0.2 |
| **Colon** | 9.1 | 7.7 | 6.5 | 5.5 | 4.7 | 3.4 | | 3.3 | 3.2 | | | 2.7 | 2.0 | | | 1.2 |
| **Liver** | 2.5 | 2.1 | 1.7 | 1.5 | 1.3 | 0.9 | | 0.9 | 0.8 | | | 0.7 | 0.4 | | | 0.2 |
| **Lung** | 17.7 | 14.7 | 12.2 | 10.1 | 8.4 | 6.0 | | 6.0 | 5.8 | | | 5.2 | 4.0 | | | 2.3 |
| **Prostate** | 0.9 | 0.8 | 0.7 | 0.6 | 0.5 | 0.4 | | 0.3 | 0.4 | | | 0.4 | 0.4 | | | 0.3 |
| **Bladder** | 2.5 | 2.1 | 1.8 | 1.5 | 1.3 | 0.9 | | 0.9 | 0.9 | | | 0.9 | 0.8 | | | 0.6 |
| **Other** | 22.3 | 14.2 | 11.1 | 9.0 | 7.5 | 5.2 | | 4.9 | 4.3 | | | 3.2 | 2.0 | | | 0.9 |
| **All solids** | 57.3 | 43.5 | 35.7 | 29.7 | 24.7 | 17.7 | | 17.3 | 16.1 | | | 13.7 | 10.1 | | | 5.7 |
| **Leukemia** | 4.0 | 4.0 | 4.0 | 3.9 | 3.7 | 3.6 | | 3.7 | 4.0 | | | 4.1 | 3.8 | | | 2.8 |
| **All cancers** | 61.2 | 47.5 | 39.7 | 33.6 | 28.5 | 21.2 | | 21.0 | 20.1 | | | 17.8 | 13.9 | | | 8.5 |
|  |  |  |  |  |  |  | |  |  | | |  |  | | |  |
| **Female** |  |  |  |  |  |  | |  |  | | |  |  | | |  |
| **Stomach** | 3.2 | 2.7 | 2.3 | 1.9 | 1.6 | 1.2 | | 1.1 | 1.1 | | | 0.9 | 0.7 | | | 0.4 |
| **Colon** | 5.7 | 4.8 | 4.1 | 3.5 | 3.0 | 2.1 | | 2.1 | 2.0 | | | 1.7 | 1.4 | | | 0.8 |
| **Liver** | 1.3 | 1.1 | 0.9 | 0.8 | 0.7 | 0.5 | | 0.4 | 0.4 | | | 0.4 | 0.3 | | | 0.2 |
| **Lung** | 35.8 | 29.8 | 24.6 | 20.4 | 17.0 | 11.9 | | 11.8 | 11.4 | | | 10.2 | 7.8 | | | 4.5 |
| **Breast** | 15.3 | 11.9 | 9.3 | 7.2 | 5.6 | 3.4 | | 2.0 | 1.1 | | | 0.5 | 0.3 | | | 0.1 |
| **Uterus** | 0.6 | 0.6 | 0.4 | 0.4 | 0.3 | 0.2 | | 0.2 | 0.2 | | | 0.2 | 0.1 | | | 0.1 |
| **Ovary** | 3.1 | 48.9 | 2.2 | 1.9 | 1.6 | 1.1 | | 1.1 | 1.0 | | | 0.8 | 0.6 | | | 0.3 |
| **Bladder** | 3.3 | 2.8 | 2.4 | 2.0 | 1.7 | 1.3 | | 1.3 | 1.2 | | | 1.2 | 1.1 | | | 0.7 |
| **Other** | 27.4 | 16.0 | 12.3 | 10.0 | 8.2 | 5.7 | | 5.4 | 4.8 | | | 3.8 | 2.6 | | | 1.3 |
| **All solids** | 95.7 | 72.2 | 58.6 | 48.0 | 39.6 | 27.4 | | 25.4 | 23.1 | | | 19.7 | 14.8 | | | 8.5 |
| **Leukemia** | 3.0 | 2.9 | 3.0 | 2.9 | 2.8 | 2.8 | | 2.9 | 3.0 | | | 3.1 | 2.9 | | | 2.1 |
| **All cancers** | 98.6 | 75.0 | 61.5 | 50.9 | 42.5 | 30.2 | | 28.2 | 26.1 | | | 22.8 | 17.7 | | | 10.6 |

**The values of lifetime risk of various cancers (in 100000 people) induced by annual BRs in Likak city**

| **Age at exposure time (year)** | | | | | | | | | | | |
| --- | --- | --- | --- | --- | --- | --- | --- | --- | --- | --- | --- |
|  | **0** | **5** | **10** | **15** | **20** | **30** | **40** | **50** | **60** | **70** | **80** |
| **Male** |  |  |  |  |  |  |  |  |  |  |  |
| **Stomach** | 5.0 | 4.3 | 3.6 | 3.0 | 2.6 | 1.8 | 1.8 | 1.6 | 1.3 | 0.9 | 0.5 |
| **Colon** | 22.1 | 18.7 | 15.8 | 13.4 | 11.4 | 8.2 | 8.0 | 7.4 | 6.2 | 4.3 | 2.0 |
| **Liver** | 4.0 | 3.3 | 2.8 | 2.4 | 2.0 | 1.4 | 1.4 | 1.2 | 0.9 | 0.5 | 0.2 |
| **Lung** | 20.6 | 17.2 | 14.2 | 11.8 | 9.8 | 6.9 | 6.8 | 6.6 | 5.8 | 4.3 | 2.2 |
| **Prostate** | 6.1 | 5.3 | 4.4 | 3.7 | 3.2 | 2.3 | 2.3 | 2.2 | 1.7 | 0.9 | 0.3 |
| **Bladder** | 13.7 | 11.6 | 9.9 | 8.3 | 7.1 | 5.2 | 5.2 | 5.0 | 4.3 | 3.1 | 1.5 |
| **Other** | 73.8 | 44.2 | 33.1 | 25.9 | 20.5 | 13.0 | 11.3 | 9.2 | 6.4 | 3.7 | 1.5 |
| **Thyroid** | 7.6 | 5.0 | 3.3 | 2.2 | 1.4 | 0.6 | 0.2 | 0.1 | 0.0 | 0.0 | 0.0 |
| **All solids** | 152.9 | 109.5 | 87.1 | 70.7 | 57.9 | 39.6 | 37.1 | 33.3 | 26.7 | 17.7 | 8.3 |
| **Leukemia** | 15.6 | 9.8 | 7.9 | 6.9 | 6.3 | 5.5 | 5.5 | 5.5 | 5.4 | 4.8 | 3.2 |
| **All cancers** | 168.4 | 119.3 | 95.0 | 77.7 | 64.2 | 45.1 | 42.6 | 38.8 | 32.1 | 22.5 | 11.4 |
|  |  |  |  |  |  |  |  |  |  |  |  |
| **Female** |  |  |  |  |  |  |  |  |  |  |  |
| **Stomach** | 6.6 | 5.6 | 4.7 | 4.0 | 3.4 | 2.4 | 2.3 | 2.1 | 1.8 | 1.2 | 0.7 |
| **Colon** | 14.5 | 12.3 | 10.4 | 8.8 | 7.5 | 5.4 | 5.2 | 4.8 | 4.1 | 3.0 | 1.5 |
| **Liver** | 1.8 | 1.5 | 1.3 | 1.1 | 0.9 | 0.7 | 0.7 | 0.6 | 0.5 | 0.3 | 0.1 |
| **Lung** | 48.2 | 40.0 | 33.1 | 27.4 | 22.7 | 15.9 | 15.8 | 15.1 | 13.2 | 9.7 | 5.1 |
| **Breast** | 77.0 | 60.1 | 46.8 | 36.3 | 28.2 | 16.6 | 9.3 | 4.6 | 2.0 | 0.8 | 0.3 |
| **Uterus** | 3.3 | 2.8 | 2.4 | 2.0 | 1.7 | 1.2 | 1.1 | 0.9 | 0.6 | 0.3 | 0.1 |
| **Ovary** | 6.8 | 90.5 | 4.8 | 3.9 | 3.3 | 2.2 | 2.0 | 1.6 | 1.2 | 0.7 | 0.3 |
| **Bladder** | 13.9 | 11.8 | 10.0 | 8.5 | 7.2 | 5.2 | 5.1 | 4.9 | 4.2 | 3.1 | 1.6 |
| **Other** | 88.0 | 47.2 | 34.4 | 26.9 | 21.2 | 13.6 | 11.9 | 9.7 | 7.2 | 4.5 | 2.0 |
| **Thyroid** | 41.7 | 27.5 | 18.1 | 11.7 | 7.4 | 2.7 | 0.9 | 0.3 | 0.1 | 0.0 | 0.0 |
| **All solids** | 301.8 | 214.6 | 165.9 | 130.6 | 103.5 | 65.8 | 54.1 | 44.6 | 34.8 | 23.5 | 11.6 |
| **Leukemia** | 12.2 | 7.4 | 5.7 | 5.0 | 4.7 | 4.1 | 4.1 | 4.1 | 3.7 | 3.4 | 2.4 |
| **All cancers** | 313.9 | 221.9 | 171.6 | 135.6 | 108.2 | 70.0 | 58.2 | 48.6 | 38.5 | 26.9 | 14.1 |

**The values of lifetime risk of various cancers mortalities (in 100000 people) induced by annual BRs in Likak city**

| **Age at exposure time (year)** | | | | | | | | | | | | | | | | |
| --- | --- | --- | --- | --- | --- | --- | --- | --- | --- | --- | --- | --- | --- | --- | --- | --- |
|  | **0** | **5** | **10** | **15** | **20** | | **30** | **40** | | **50** | **60** | | | **70** | **80** | |
| **Male** |  |  |  |  |  | |  |  | |  |  | | |  |  | |
| **Stomach** | 2.7 | 2.2 | 2.0 | 1.6 | 1.4 | 1.1 | | 1.0 | 0.9 | | | 0.7 | 0.5 | | | 0.3 |
| **Colon** | 10.7 | 9.1 | 7.7 | 6.5 | 5.5 | 4.0 | | 3.9 | 3.7 | | | 3.2 | 2.4 | | | 1.4 |
| **Liver** | 2.9 | 2.4 | 2.0 | 1.8 | 1.5 | 1.1 | | 1.1 | 0.9 | | | 0.8 | 0.5 | | | 0.3 |
| **Lung** | 20.9 | 17.3 | 14.4 | 12.0 | 9.9 | 7.0 | | 7.0 | 6.8 | | | 6.1 | 4.7 | | | 2.8 |
| **Prostate** | 1.1 | 1.0 | 0.8 | 0.7 | 0.6 | 0.5 | | 0.4 | 0.5 | | | 0.5 | 0.5 | | | 0.3 |
| **Bladder** | 3.0 | 2.5 | 2.1 | 1.8 | 1.5 | 1.1 | | 1.1 | 1.1 | | | 1.1 | 1.0 | | | 0.7 |
| **Other** | 26.3 | 16.8 | 13.1 | 10.6 | 8.8 | 6.2 | | 5.8 | 5.1 | | | 3.8 | 2.4 | | | 1.1 |
| **All solids** | 67.6 | 51.3 | 42.1 | 35.0 | 29.2 | 20.8 | | 20.4 | 19.0 | | | 16.2 | 11.9 | | | 6.7 |
| **Leukemia** | 4.7 | 4.7 | 4.7 | 4.6 | 4.4 | 4.2 | | 4.4 | 4.7 | | | 4.8 | 4.5 | | | 3.4 |
| **All cancers** | 72.2 | 56.0 | 46.8 | 39.6 | 33.6 | 25.0 | | 24.8 | 23.7 | | | 21.0 | 16.4 | | | 10.1 |
|  |  |  |  |  |  |  | |  |  | | |  |  | | |  |
| **Female** |  |  |  |  |  |  | |  |  | | |  |  | | |  |
| **Stomach** | 3.7 | 3.2 | 2.7 | 2.2 | 1.9 | 1.4 | | 1.3 | 1.2 | | | 1.1 | 0.9 | | | 0.5 |
| **Colon** | 6.7 | 5.7 | 4.8 | 4.1 | 3.5 | 2.5 | | 2.4 | 2.3 | | | 2.0 | 1.6 | | | 1.0 |
| **Liver** | 1.6 | 1.3 | 1.1 | 0.9 | 0.8 | 0.6 | | 0.5 | 0.5 | | | 0.5 | 0.3 | | | 0.2 |
| **Lung** | 42.3 | 35.1 | 29.0 | 24.1 | 20.0 | 14.0 | | 13.9 | 13.4 | | | 12.0 | 9.2 | | | 5.3 |
| **Breast** | 18.0 | 14.1 | 11.0 | 8.5 | 6.6 | 4.0 | | 2.3 | 1.2 | | | 0.6 | 0.3 | | | 0.1 |
| **Uterus** | 0.7 | 0.7 | 0.5 | 0.5 | 0.4 | 0.3 | | 0.3 | 0.2 | | | 0.2 | 0.1 | | | 0.1 |
| **Ovary** | 3.6 | 48.9 | 2.6 | 2.2 | 1.8 | 1.3 | | 1.3 | 1.2 | | | 1.0 | 0.7 | | | 0.3 |
| **Bladder** | 3.9 | 3.4 | 2.8 | 2.4 | 2.0 | 1.5 | | 1.5 | 1.4 | | | 1.4 | 1.2 | | | 0.9 |
| **Other** | 32.3 | 18.9 | 14.5 | 11.8 | 9.7 | 6.8 | | 6.4 | 5.7 | | | 4.5 | 3.1 | | | 1.6 |
| **All solids** | 112.8 | 85.1 | 69.1 | 56.6 | 46.7 | 32.3 | | 29.9 | 27.3 | | | 23.3 | 17.4 | | | 10.0 |
| **Leukemia** | 3.5 | 3.4 | 3.5 | 3.4 | 3.4 | 3.4 | | 3.4 | 3.5 | | | 3.6 | 3.4 | | | 2.5 |
| **All cancers** | 116.3 | 88.5 | 72.5 | 60.1 | 50.1 | 35.6 | | 33.3 | 30.8 | | | 26.9 | 20.8 | | | 12.5 |

**The values of lifetime risk of various cancers (in 100000 people) induced by annual BRs in Landeh city**

| **Age at exposure time (year)** | | | | | | | | | | | | |
| --- | --- | --- | --- | --- | --- | --- | --- | --- | --- | --- | --- | --- |
|  | **0** | **5** | **10** | **15** | **20** | **30** | **40** | **50** | **60** | **70** | **80** |  |
| **Male** |  |  |  |  |  |  |  |  |  |  |  |  |
| **Stomach** | 4.1 | 3.5 | 2.9 | 2.5 | 2.1 | 1.5 | 1.4 | 1.3 | 1.1 | 0.8 | 0.4 |  |
| **Colon** | 18.0 | 15.3 | 12.9 | 10.9 | 9.3 | 6.7 | 6.5 | 6.1 | 5.0 | 3.5 | 1.6 |  |
| **Liver** | 3.3 | 2.7 | 2.3 | 1.9 | 1.6 | 1.2 | 1.1 | 1.0 | 0.8 | 0.4 | 0.2 |  |
| **Lung** | 16.8 | 14.0 | 11.6 | 9.6 | 8.0 | 5.6 | 5.6 | 5.4 | 4.8 | 3.5 | 1.8 |  |
| **Prostate** | 5.0 | 4.3 | 3.6 | 3.1 | 2.6 | 1.9 | 1.9 | 1.8 | 1.4 | 0.8 | 0.3 |  |
| **Bladder** | 11.2 | 9.5 | 8.0 | 6.8 | 5.8 | 4.2 | 4.2 | 4.1 | 3.5 | 2.5 | 1.2 |  |
| **Other** | 60.2 | 36.0 | 26.9 | 21.1 | 16.7 | 10.6 | 9.2 | 7.5 | 5.3 | 3.1 | 1.2 |  |
| **Thyroid** | 6.2 | 4.1 | 2.7 | 1.8 | 1.1 | 0.5 | 0.2 | 0.1 | 0.0 | 0.0 | 0.0 |  |
| **All solids** | 124.6 | 89.3 | 71.0 | 57.6 | 47.2 | 32.3 | 30.2 | 27.2 | 21.8 | 14.5 | 6.8 |  |
| **Leukemia** | 12.7 | 8.0 | 6.4 | 5.6 | 5.1 | 4.5 | 4.5 | 4.5 | 4.4 | 3.9 | 2.6 |  |
| **All cancers** | 137.3 | 97.3 | 77.4 | 63.3 | 52.3 | 36.8 | 34.7 | 31.7 | 26.2 | 18.4 | 9.3 |  |
|  |  |  |  |  |  |  |  |  |  |  |  |  |
| **Female** |  |  |  |  |  |  |  |  |  |  |  |  |
| **Stomach** | 5.4 | 4.6 | 3.9 | 3.3 | 2.8 | 1.9 | 1.9 | 1.7 | 1.4 | 1.0 | 0.6 |  |
| **Colon** | 11.8 | 10.0 | 8.5 | 7.2 | 6.1 | 4.4 | 4.2 | 3.9 | 3.3 | 2.4 | 1.2 |  |
| **Liver** | 1.5 | 1.2 | 1.1 | 0.9 | 0.8 | 0.5 | 0.5 | 0.5 | 0.4 | 0.3 | 0.1 |  |
| **Lung** | 39.3 | 32.6 | 27.0 | 22.3 | 18.5 | 13.0 | 12.9 | 12.3 | 10.8 | 7.9 | 4.1 |  |
| **Breast** | 62.7 | 49.0 | 38.1 | 29.6 | 23.0 | 13.6 | 7.6 | 3.8 | 1.7 | 0.6 | 0.2 |  |
| **Uterus** | 2.7 | 2.3 | 1.9 | 1.6 | 1.4 | 1.0 | 0.9 | 0.7 | 0.5 | 0.3 | 0.1 |  |
| **Ovary** | 5.6 | 90.5 | 3.9 | 3.2 | 2.7 | 1.8 | 1.7 | 1.3 | 1.0 | 0.6 | 0.3 |  |
| **Bladder** | 11.4 | 9.6 | 8.1 | 6.9 | 5.8 | 4.2 | 4.2 | 4.0 | 3.4 | 2.5 | 1.3 |  |
| **Other** | 71.7 | 38.5 | 28.0 | 21.9 | 17.3 | 11.1 | 9.7 | 7.9 | 5.8 | 3.6 | 1.6 |  |
| **Thyroid** | 34.0 | 22.4 | 14.7 | 9.5 | 6.1 | 2.2 | 0.8 | 0.2 | 0.1 | 0.0 | 0.0 |  |
| **All solids** | 246.0 | 174.9 | 135.3 | 106.5 | 84.4 | 53.7 | 44.1 | 36.3 | 28.3 | 19.2 | 9.5 |  |
| **Leukemia** | 9.9 | 6.0 | 4.6 | 4.1 | 3.8 | 3.4 | 3.3 | 3.3 | 3.1 | 2.7 | 2.0 |  |
| **All cancers** | 255.9 | 180.9 | 139.9 | 110.6 | 88.2 | 57.1 | 47.5 | 39.6 | 31.4 | 21.9 | 11.5 |  |

**The values of lifetime risk of various cancers mortalities (in 100000 people) induced by annual BRs in Landeh city**

| **Age at exposure time (year)** | | | | | | | | | | | | | | | | |
| --- | --- | --- | --- | --- | --- | --- | --- | --- | --- | --- | --- | --- | --- | --- | --- | --- |
|  | **0** | **5** | **10** | **15** | **20** | | **30** | **40** | | **50** | **60** | | | **70** | **80** | |
| **Male** |  |  |  |  |  | |  |  | |  |  | | |  |  | |
| **Stomach** | 2.2 | 1.8 | 1.6 | 1.3 | 1.1 | 0.9 | | 0.8 | 0.7 | | | 0.6 | 0.4 | | | 0.2 |
| **Colon** | 8.7 | 7.4 | 6.3 | 5.3 | 4.5 | 3.3 | | 3.2 | 3.1 | | | 2.6 | 1.9 | | | 1.1 |
| **Liver** | 2.4 | 2.0 | 1.7 | 1.4 | 1.2 | 0.9 | | 0.9 | 0.8 | | | 0.6 | 0.4 | | | 0.2 |
| **Lung** | 17.0 | 14.1 | 11.7 | 9.8 | 8.1 | 5.7 | | 5.7 | 5.6 | | | 5.0 | 3.8 | | | 2.3 |
| **Prostate** | 0.9 | 0.8 | 0.6 | 0.5 | 0.5 | 0.4 | | 0.3 | 0.4 | | | 0.4 | 0.4 | | | 0.3 |
| **Bladder** | 2.4 | 2.0 | 1.7 | 1.4 | 1.2 | 0.9 | | 0.9 | 0.9 | | | 0.9 | 0.8 | | | 0.5 |
| **Other** | 21.4 | 13.7 | 10.7 | 8.7 | 7.2 | 5.0 | | 4.7 | 4.1 | | | 3.1 | 1.9 | | | 0.9 |
| **All solids** | 55.1 | 41.8 | 34.3 | 28.6 | 23.8 | 17.0 | | 16.6 | 15.5 | | | 13.2 | 9.7 | | | 5.5 |
| **Leukemia** | 3.8 | 3.8 | 3.8 | 3.8 | 3.6 | 3.4 | | 3.6 | 3.8 | | | 3.9 | 3.7 | | | 2.7 |
| **All cancers** | 58.9 | 45.6 | 38.1 | 32.3 | 27.4 | 20.4 | | 20.2 | 19.3 | | | 17.1 | 13.4 | | | 8.2 |
|  |  |  |  |  |  |  | |  |  | | |  |  | | |  |
| **Female** |  |  |  |  |  |  | |  |  | | |  |  | | |  |
| **Stomach** | 3.1 | 2.6 | 2.2 | 1.8 | 1.6 | 1.1 | | 1.1 | 1.0 | | | 0.9 | 0.7 | | | 0.4 |
| **Colon** | 5.5 | 4.6 | 3.9 | 3.3 | 2.8 | 2.0 | | 2.0 | 1.9 | | | 1.7 | 1.3 | | | 0.8 |
| **Liver** | 1.3 | 1.1 | 0.9 | 0.8 | 0.6 | 0.5 | | 0.4 | 0.4 | | | 0.4 | 0.3 | | | 0.2 |
| **Lung** | 34.4 | 28.6 | 23.7 | 19.7 | 16.3 | 11.4 | | 11.4 | 10.9 | | | 9.8 | 7.5 | | | 4.3 |
| **Breast** | 14.7 | 11.5 | 8.9 | 7.0 | 5.4 | 3.3 | | 1.9 | 1.0 | | | 0.5 | 0.3 | | | 0.1 |
| **Uterus** | 0.6 | 0.5 | 0.4 | 0.4 | 0.3 | 0.2 | | 0.2 | 0.2 | | | 0.2 | 0.1 | | | 0.1 |
| **Ovary** | 2.9 | 48.9 | 2.1 | 1.8 | 1.5 | 1.1 | | 1.1 | 1.0 | | | 0.8 | 0.5 | | | 0.3 |
| **Bladder** | 3.2 | 2.7 | 2.3 | 1.9 | 1.7 | 1.2 | | 1.2 | 1.2 | | | 1.2 | 1.0 | | | 0.7 |
| **Other** | 26.3 | 15.4 | 11.8 | 9.6 | 7.9 | 5.5 | | 5.2 | 4.6 | | | 3.7 | 2.5 | | | 1.3 |
| **All solids** | 92.0 | 69.4 | 56.3 | 46.2 | 38.1 | 26.3 | | 24.4 | 22.2 | | | 19.0 | 14.2 | | | 8.1 |
| **Leukemia** | 2.8 | 2.8 | 2.8 | 2.8 | 2.7 | 2.7 | | 2.8 | 2.9 | | | 2.9 | 2.8 | | | 2.0 |
| **All cancers** | 94.8 | 72.2 | 59.1 | 49.0 | 40.8 | 29.0 | | 27.2 | 25.1 | | | 21.9 | 17.0 | | | 10.2 |
